# Two‐ and three‐dimensional in vitro nucleus pulposus cultures: An in silico analysis of local nutrient microenvironments

**DOI:** 10.1002/jsp2.1222

**Published:** 2022-08-30

**Authors:** Emily E. McDonnell, Conor T. Buckley

**Affiliations:** ^1^ Trinity Centre for Biomedical Engineering, Trinity Biomedical Sciences Institute, Trinity College Dublin The University of Dublin Dublin Ireland; ^2^ Discipline of Mechanical, Manufacturing and Biomedical Engineering, School of Engineering, Trinity College Dublin The University of Dublin Dublin Ireland; ^3^ Advanced Materials and Bioengineering Research (AMBER) Centre, Royal College of Surgeons in Ireland & Trinity College Dublin The University of Dublin Dublin Ireland; ^4^ Tissue Engineering Research Group, Department of Anatomy and Regenerative Medicine Royal College of Surgeons in Ireland Dublin Ireland

**Keywords:** cell culture, glucose, in silico, microenvironment, oxygen, pH

## Abstract

**Background:**

It is well established that the unique biochemical microenvironment of the intervertebral disc plays a predominant role in cell viability and biosynthesis. However, unless the effect of microenvironmental conditions is primary to a study objective, in vitro culture parameters that are critical for reproducibility are both varied and not routinely reported.

**Aims:**

This work aims to investigate the local microenvironments of commonly used culture configurations, highlighting physiological relevance, potential discrepancies, and elucidating possible heterogeneity across the research field.

**Materials and Methods:**

This work uses nutrient‐transport in silico models to reflect on the effect of often underappreciated parameters, such as culture geometry and diffusional distance (vessel, media volume, construct size), seeding density, and external boundary conditions on the local microenvironment of two‐dimensional (2D) and three‐dimensional (3D) in vitro culture systems.

**Results:**

We elucidate important discrepancies between the external boundary conditions such as the incubator level or media concentrations and the actual local cellular concentrations. Oxygen concentration and cell seeding density were found to be highly influential parameters and require utmost consideration when utilizing 3D culture systems.

**Discussion:**

This work highlights that large variations in the local nutrient microenvironment can easily be established without consideration of several key parameters. Without careful deliberation of the microenvironment within each specific and unique system, there is the potential to confound in vitro results leading to heterogeneous results across the research field in terms of biosynthesis and matrix composition.

**Conclusion:**

Overall, this calls for a greater appreciation of key parameters when designing in vitro experiments. Better harmony and standardization of physiologically relevant local microenvironments are needed to push toward reproducibility and successful translation of findings across the research field.

## Abstract 2

Large variations in the local nutrient microenvironment can easily be established without careful consideration of several key parameters. While one external concentration may be suitable for one culture configuration, they may not be appropriate for another. External conditions need to be tailored to the specific cells and culture system to establish homogeneous and physiologically relevant microenvironments. Taken together, more deliberate consideration of the external boundary concentrations and in vitro culture design, harmony and standardization of a physiologically relevant microenvironment will push toward greater reproducibility and more successful translation of findings across the field.

## 1 INTRODUCTION

It is well established that the unique biochemical microenvironment of the intervertebral disc (IVD) has a predominant role in degeneration and the success of potential regenerative strategies.^1^, ^2^, ^3^ Due to the IVD being avascular, nutrients, and metabolites must be transported to and from the cells through the extracellular matrix (ECM), giving rise to gradients throughout the tissue.^4^ The viability and biosynthesis of a sparse population of central nucleus pulposus (NP) cells are critical to the maintenance of a highly specific ECM composition required for the inherent biomechanical function of the IVD. As a result, the effect of nutrient concentrations and pH on native NP cells,^5^, ^6^, ^7^, ^8^, ^9^, ^10^, ^11^, ^12^, ^13^, ^14^ and potential cell therapies has been extensively investigated.^15^, ^16^, ^17^, ^18^, ^19^ Key findings suggest that glucose is the nutrient critical for maintaining viability, with bovine NP cell death occurring when glucose falls below ~0.5 mM for more than 3 days, while cells have remained viable up to 13 days in the absence of oxygen.^6^, ^7^ However, oxygen level appears to play a dominant role in maintaining the NP phenotype and controlling the synthesis of key water‐binding proteoglycan (PG) molecules of the ECM.^20^, ^21^ Meanwhile low pH due to the accumulation of lactic acid, has long been connected with induced cell death and hampered PG synthesis,^5^, ^7^, ^8^, ^22^ but more recently it has also been associated with the upregulation of proinflammatory cytokines and pain‐related neurogenic factors.^23^ As a result, it is evident that for repeatable and clinically translatable in vitro results, experiments need to be performed at consistent and physiologically relevant levels of nutrition.

**FIGURE 1:**
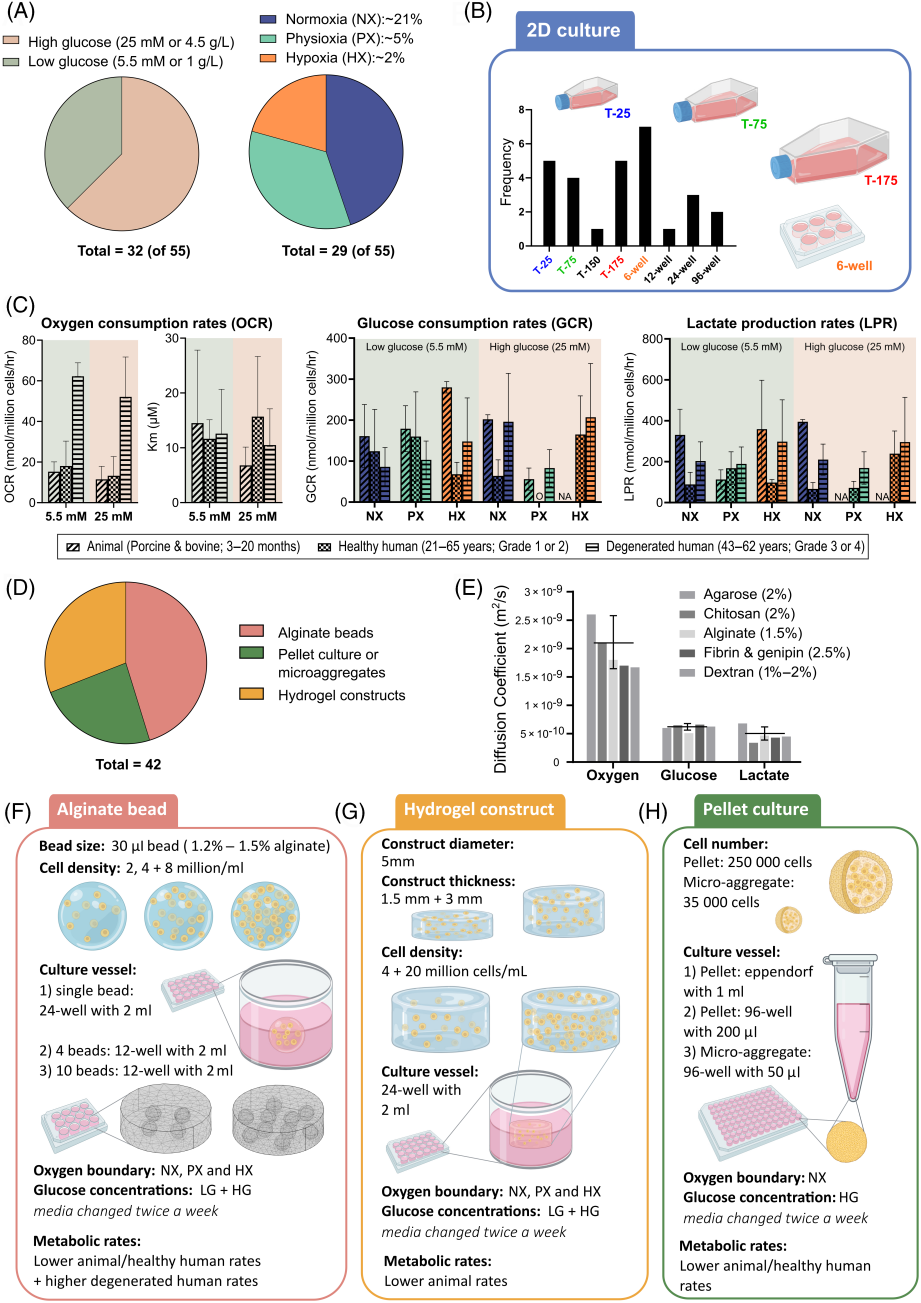
(A) The most used glucose concentrations and incubator oxygen levels across 55 reviewed studies. (B) The frequency of culture vessel used across these studies for 2D NP cell expansion and culture. (C) Compiled metabolic rates gathered from the literature and graphed according to glucose concentration and external oxygen concentration. (D) Most commonly used 3D culture system for NP cells across 42 studies. (E) Effective diffusion coefficients for oxygen, glucose, and lactate through several relevant hydrogels at 37°C. (F–H) Most common configurations (geometrical dimensions, cell density, boundary concentrations, and culture vessels) for alginate beads, cylindrical hydrogel constructs, and pellet culture

Our recent study sought to consolidate the current knowledge of the IVD nutrient microenvironment and re‐evaluate the concentrations in the context of different stages of degeneration.^24^ This work suggests that at a stage of degeneration when cell‐based regeneration remains a viable treatment option, the central NP microenvironment consists of glucose concentrations of approximately 1–3.5 mM, an average oxygen level of 6–8 %O_2_, and a median pH of 7. Therefore, when investigating the response of potential cell therapies in vitro, we need to tailor the biochemical microenvironments to these values to ensure that results are more physiologically relevant and clinically translatable. This concept has recently been deliberated for more advanced ex vivo disc organ culture systems,^25^ while it is often considered that in less complex in vitro cell culture, these conditions can be simply implemented through culturing cells in low oxygen, reduced glucose and/or serum and increased acidity. However, aside from studies which specifically study the effect of microenvironmental conditions, the majority of disc cell culture across the research field do not adjust the pH of the culture media. Glucose concentration of the culture media and the incubator oxygen levels are more commonly regarded as controllable in vitro boundary conditions. Despite this, only 32 (58.2%) of 55 reviewed papers reported on the glucose concentration and only 23 (41.78%) reported on incubator oxygen level two‐dimensional (2D) cell expansion. Figure 1A shows that among the studies that reported on the boundary nutrient concentrations 62.5% used high glucose (HG: 25 mM or 4.5 g/L) and 37.5% used low glucose (LG: 5.5 mM or 1 g/L), while 44.8% used “normoxia” (NX: 20–21 %O_2_), 34.5% used “physioxia” (PX: ~5 %O_2_), and 20.7% used “hypoxia” (HX: ~2 %O_2_). Taking note that of the 23 studies reporting oxygen values, 6 studies investigated more than one oxygen level. This not only highlights large variation across the field but also that parameters that are critical for reproducibility are not routinely being reported. There is an urgency for this to be addressed, particularly with a recent push toward harmonization within disc research and the increased attention to the reproducibility of research findings across all scientific fields.^26^

Furthermore, a disconnect often occurs between the external incubator or media concentrations and the actual local cellular concentrations.^27^, ^28^, ^29^, ^30^ There is an underappreciation for the effects of parameters such as diffusion rate, media volume and cell density on the true nutrient microenvironment of cell culture systems. A number of studies use soft scaffolds such as hydrogels to mimic the native disc tissue and maintain cells in their three‐dimensional (3D) phenotype. However, potential nutrient gradients through these systems will not only affect cell viability and differentiation but also regulate gene expression and metabolism, creating distinct regions of ECM deposition. Therefore, it is critical that we carefully consider the combined effects key culture parameters may have on the microenvironment of in vitro systems and the downstream confounding influence on heterogenous matrix synthesis. This work aims to characterize the local nutrient microenvironment of 2D cell monolayers and commonly used 3D in vitro culture systems, to highlight the effect of culturing parameters and to place “standard practice” culturing conditions into context in terms of physiological relevance.

## 2 METHODS

Long‐term leaders in disc cell culture were identified through the ORS Spine subgroup involved in standardization and harmonization of cell isolation and culture methods. As a result, we looked at studies over the last ~15 years from 20 prominent disc groups from across 14 different universities/medical centers. A list of the reviewed literature with a summary of key parameters can be found in Table S1.

### 2.1 Two‐dimensional cell culture models

Figure 1B highlights the frequency of different cell culture vessels used for 2D monolayer cell culture and expansion. The in silico nutrient transport models were created using COMSOL Multiphysics 6 (COMSOL Inc., Burlington, Massachusetts) for the most used culture vessels. Geometries were created based on Corning™ Costar™ culture plates and SARSTEDT flasks with a standard working volume of media (6‐well = 2 ml, T‐25 = 5 ml, T‐75 = 10 ml, and T‐175 = 20 ml) and an effective diffusion coefficient of 2.8 × 10^−9^ m^2^/s for oxygen, 5.67 × 10^−10^ m^2^/s for glucose, and 5.68 × 10^−10^ m^2^/s for lactate at 37°C.^31^, ^32^, ^33^ Nutrient concentrations at the cell surface were established using a transient analysis of coupled reaction–diffusion equations together with cell proliferation kinetics.

### 2.2 Metabolic rates and proliferation kinetics

Several studies have reported oxygen consumption rates (OCR),^8^, ^9^, ^10^, ^12^ glucose consumption rates (GCR), and lactate production rates (LPR) for NP cells from animals and humans under varying nutrient conditions.^8^, ^10^, ^13^, ^14^, ^17^, ^34^, ^35^ Tabulated rates from these studies can be found in Table S2–S4. For this work the literature has been compiled based on the external boundary conditions of glucose (LG or HG) and oxygen (NX, PX, or HX), as shown in Figure 1C. Significantly higher oxygen consumption has been reported for degenerated human cells (aged 43–62 years, Thompson Grade III or IV*)*,^12^ as a result OCRs can be separated into a “lower” rate group comprising of values reported for animal and healthy human cells (aged 21–65 years, Thompson Grade I or II) and a “higher” rate group for a degenerated phenotype. However, similar categorizing of glycolytic rates does not appear to be possible with the literature available and average GCR and LPR values will be used regardless of species/degeneration stage. As previously demonstrated together with successful ex vivo validation,^25^ OCR (μM/h) is modeled as being dependent on local pH and oxygen by employing Michaelis–Menten equations.^8^, ^9^, ^36^, ^37^

$$\mathrm{OCR}=-\frac{V_{\max}\left(\mathrm{pH}-4.95\right)C^{O_{2}}}{K_{m}\left(\mathrm{pH}-4.59\right)+C^{O_{2}}}\rho_{\text{cell}}$$

where t is the time (h), $C^{O_{2}}$ is the local oxygen concentration (μM), pH is the local pH level, and $\rho_{\text{cell}}$ is the cell density (million cell/ml). $V_{\max}$ is the maximum consumption rate (nmol/million cells/h) and $K_{m}$ is the rate limiting Michaelis–Menten constant (μM).

GCR was more explicitly modeled using a maximum GCR based on the external glucose boundary conditions (as no clear and obvious difference exists between 5.5 and 25 mM in Figure 1C) but becomes rate limited at ~2 mM, by curve fitting Michaelis–Menten kinetics to experimental measurements at lower glucose concentrations (<5 mM).^14^, ^17^

$$\mathrm{GCR}=-\frac{V_{\max}\left(C^{\text{gluc}}\right)}{K_{m}+C^{\text{gluc}}}\rho_{\text{cell}}$$

where t is the time (hr), $C^{\text{gluc}}$ is the local glucose concentration (mM), $\rho_{\text{cell}}$ is the cell density (million cell/ml). $V_{\max}$ is the maximum consumption rate (nmol/million cells/h) and $K_{m}$ is the rate limiting Michaelis–Menten constant (μM).

In order to capture rate limited glycolysis, LPR was implicitly modeled based on the ratio of lac:gluc molecules which is typically 2:1.^8^, ^9^, ^36^, ^37^ However, considering the compiled experimental literature this ratio appears to vary as a function of oxygen. Furthermore, when the pH drops below 6.7 in certain culture configurations, LPR was modeled explicitly to capture experimental observations of rates dropping from ~200 nmol/million cells/h at pH 7.4 to ~150 nmol/million cells/h at pH 6.7 and to ~50 nmol/million cells/h at pH 6.2.^8^ The finalized metabolic parameters used in the models, based on the media glucose and external oxygen levels are presented in Table 1.

**TABLE 1:** Metabolic parameters used in each in silico model based on external boundary conditions and the species or degeneration stage being investigated

LG + varying oxygen	LG + NX	LG + PX	LG + HX
OCR (*V*_max_); Units: nmol/million cells/h	Animal + healthy human: 17 Degenerated human: 62		
Rate limiting oxygen (*K*_*m*_); Units: μM	12		
GCR; Units: nmol/million cells/h	143	103	165
Rate limiting glucose (*K*_*m*_); Units: mM	2		
LPR; Units: nmol/million cells/h	207	168	296
Ratio (lac:gluc)	1.4:1	1.6:1	1.8:1

HG + varying oxygen	HG + NX	HG + PX	HG + HX
OCR (*V*_max_); Units: nmol/million cells/h	Animal + healthy human: 12 Degenerated human: 52		
Rate limiting oxygen (*K*_*m*_); Units: μM	12		
GCR; Units: nmol/million cells/h	143	103	165
Rate limiting glucose (*K*_*m*_); Units: mM	2		
LPR; Units: nmol/million cells/h	207	168	296
Ratio (lac:gluc)	1.4:1	1.6:1	1.8:1

Abbreviations: GCR, glucose consumption rate; HG, high glucose; HX, hypoxia; LG, low glucose; LPR, lactate production rate; NX, normoxia; OCR, oxygen consumption rate; PX, physioxia.

Cell proliferation was modeled using first order kinetics based on our observed population doubling time (the time for the doubling of a single cell under mitosis). Based on our experience of culturing NP derived porcine cells and assuming an initial seeding density of 5 × 10^3^ cells/cm^2^ and an upper limit of 5 × 10^6^ from a T‐175 after 5–6 days (i.e., ~ 28 571 cells/cm^2^ at 80% confluency), an exponential growth rate (*k*) of 0.348 [1/*d*] can be calculated using the population doubling time.

$$N_{t}=N_{0}e^{\mathrm{kt}}$$

$$k=\frac{\ln\left(\frac{N_{t}}{N_{0}}\right)}{t}$$

where k is the frequency of cell cycles per unit time, $N_{t}$ is cell number at time t, $N_{0}$ is the initial cell number, and t is the culture time in days.

Given that it is common practice to perform a media exchange twice a week and given the assumption of 5–6 days to ~80% confluency, the transient 2D analysis was modeled for 7 days, incorporating a media exchange at the midway point. Although other studies may report different growth rates, the culture time is arbitrary; whether cells reach 80% confluency at 5–6 days or 12–15 days,^38^ the results at 80% confluency will be the same regardless of the time frame. An example of different proliferation kinetics can be found in Figure S1A–C.

### 2.3 Three‐dimensional cell culture models

Across 42 studies from prominent research groups in disc cell culture, alginate beads were the most popular 3D culture system (45.2%), followed by cylindrical hydrogel constructs (31.0%) and then pellet or microaggregate cultures (23.8%), Figure 1D. The effective diffusion coefficient of oxygen,^39^, ^40^, ^41^, ^42^ glucose,^43^, ^44^, ^45^, ^46^ and lactate,^46^, ^47^, ^48^, ^49^ through a number of different hydrogels were gathered to experimentally inform the in silico models, Figure 1E. Within the bead model alginate‐specific diffusion coefficients for oxygen (1.8 × 10^−9^ m^2^/s), glucose (5.1 × 10^−10^ m^2^/s), and lactate (4.67 × 10^−10^ m^2^/s) were used.^39^, ^43^, ^47^ The cylindrical constructs in the literature use a range of different hydrogel material types, thus an average diffusion coefficient across a number of typical hydrogel concentrations was implemented (2.1 ± 0.5 × 10^−9^ m^2^/s, 6.3 ± 0.6 × 10^−10^ m^2^/s, and 5.1 ± 1.1 × 10^−10^ m^2^/s, for oxygen, glucose and lactate, respectively). Diffusion through culture media was the same as for the 2D models. However, as an idealized model, diffusion was not modelled through the base of the construct which was in contact with the tissue culture plastic.

Hydrogel geometry, cell seeding densities, culture vessel type and media volume, external boundary concentrations, and metabolic rates all play a role in establishing the local cell nutrient microenvironment. The most utilized configurations for alginate beads, cylindrical constructs, and pellet cultures are presented in Figure 1F–H, providing a schematic for the different model iterations presented in this work. Because all possible combinations cannot be investigated within the scope of this work, a sensitivity analysis for the lowest/highest rates of metabolism reported from the literature can be found in Figure S1D.

### 2.4 External boundary concentrations: Glucose and oxygen

**FIGURE 2:**
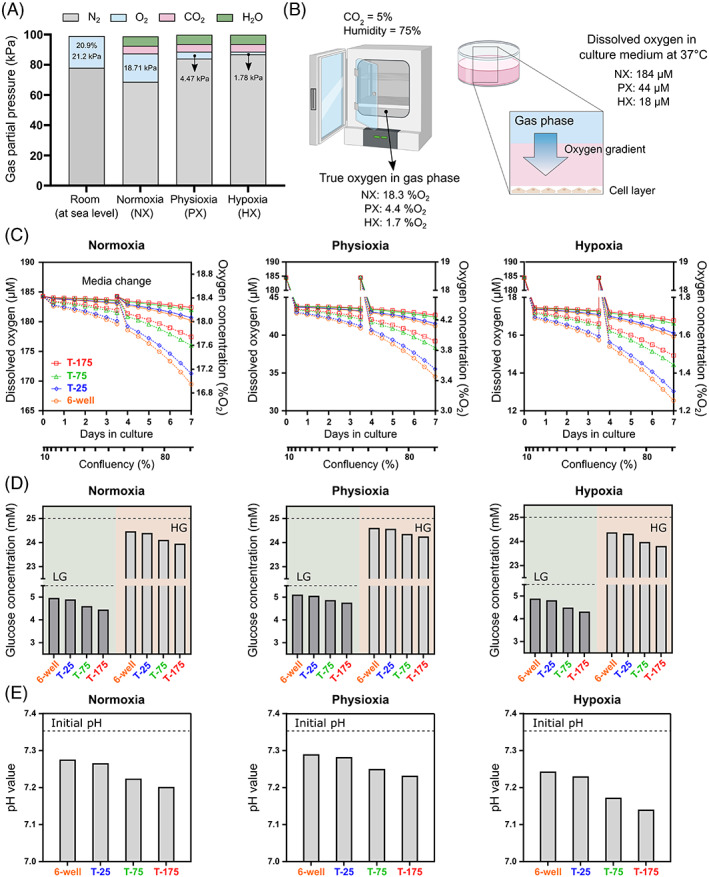
(A) The volume/volume ratio of oxygen to other gases in an incubator is decreased compared with that of dry air in a room. Values are shown for sea level. (B) As a result, the relative gas concentration in a normoxia (NX), physioxia (PX), or hypoxia (HX) incubator, with the addition of 5% CO_2_ and 75% humidity, are lower than the conventional concentrations often cited. These incubator oxygen levels can then be converted to the concentration of dissolved oxygen by using an oxygen solubility coefficient in culture media at 37°C. (C) A temporal analysis of the oxygen concentration at the cell surface for cells proliferating over a 7‐day period in either an NX, PX or HX incubator. Solid line indicates a lower animal/healthy human metabolism, dashed lines indicate a higher degenerated phenotype. (D) Glucose concentration of the media at 80% confluency for both low (5.5 mM) and high (25 mM) glucose media in a NX, PX, and HX incubator. (E) pH concentration of the media at 80% confluency in a NX, PX, and HX incubator.

As mentioned previously, culture media is typically either LG (5.5 mM) or HG (25 mM) and both concentrations will be investigated for all culture configurations except pellet culture, as all pellet literature report the use of HG only. However, in terms of oxygen the volume/volume ratio of oxygen to other gases in an incubator is decreased compared with dry room air (21.2 kPa or 159 mmHg at sea level), Figure 2A. As a result, the relative gas concentration in a NX, PX, or HX incubator, with the addition of 5% CO_2_ (38 mmHg) and 75% humidity (47 mmHg), is lower than the conventional concentrations typically cited.^50^ Furthermore, for modeling through COMSOL, the partial pressure of incubator oxygen must be converted into the concentration of dissolved oxygen by using Henry's law (oxygen solubility coefficient of 1.3 μM/mmHg in culture media at 37°C).^29^, ^50^ The resulting oxygen concentration of the culture media equilibrated within each oxygen incubator is shown in Figure 2B.

## 3 RESULTS

2D results are presented as a transient analysis to capture the effect of cell proliferation and media exchange over a 7‐day period. Figure 2C shows the oxygen concentration at the cell surface of a 6‐well plate, T‐25, T‐75, or T‐175 flask as a function of confluency in either a NX, PX, or HX incubator. As expected, oxygen decreases at the cell surface as the cells multiply creating greater demand for oxygen. However, there is no major difference in surface concentration between the culture vessels, particularly for cells categorized as having lower metabolic rates. Even at higher degenerated rates of oxygen metabolism, concentrations at the cell surface are predicted to only drop a maximum of 1.3 %O_2_ at NX, 0.78 %O_2_ at PX, and 0.43 %O_2_ at HX over the expansion period. Figure 2D shows the average glucose concentration of both LG and HG culture media at ~80% confluency (day 5–6), following a standard media exchange of twice weekly, while Figure 2E shows the corresponding pH level of the media. As expected, glucose and pH are predicted highest in the culture vessel with the lowest cell yield (6‐well) and lowest in the culture vessel with the greatest cell yield (T‐175). Additionally, no significant difference was seen in glucose between the different oxygen incubators in 2D due to a surplus of glucose, while pH reduces slightly more under HX due to increased glycolytic rates.

**FIGURE 3:**
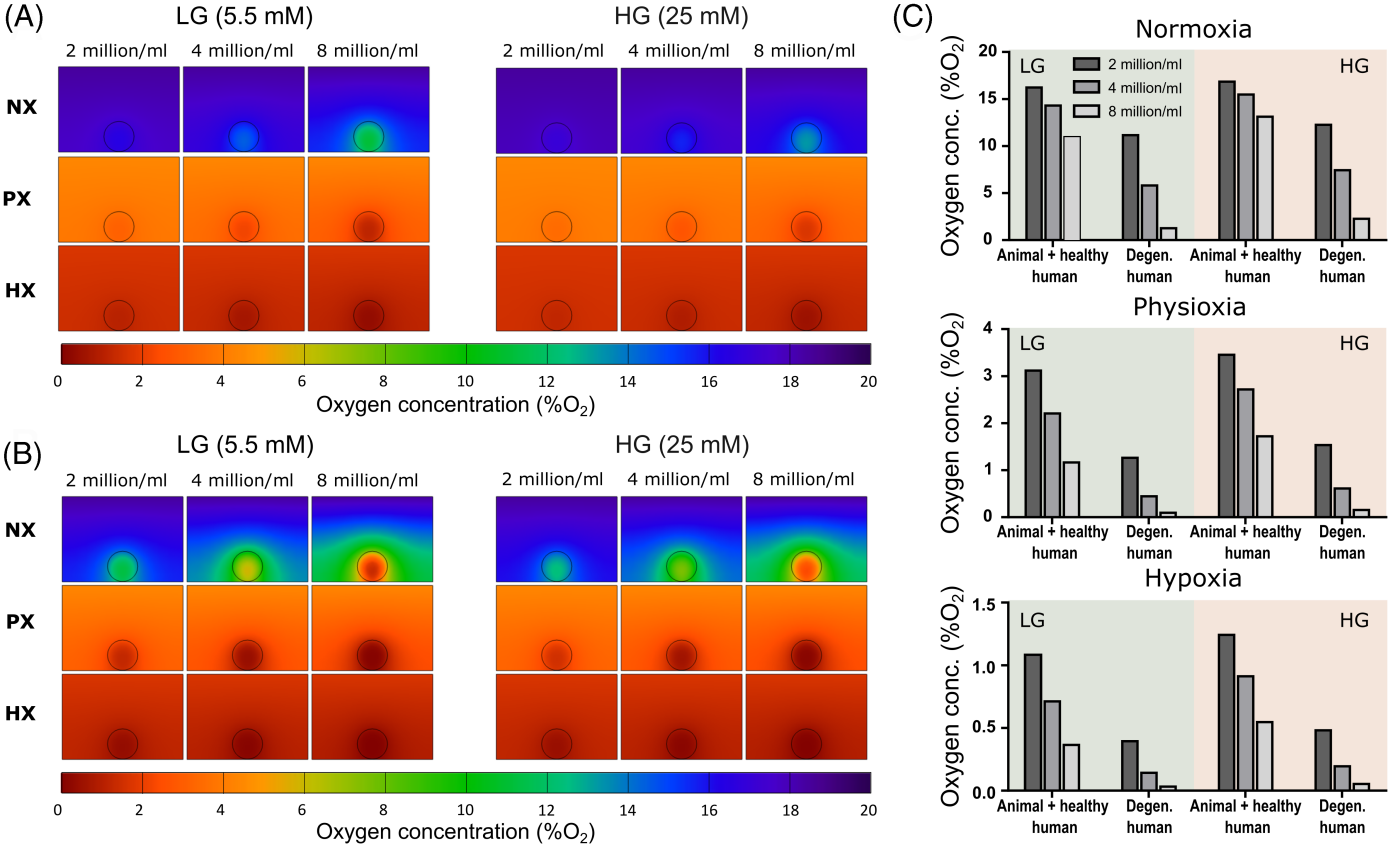
Investigating the effect of three different seeding densities on the oxygen concentration in a single 30 μl alginate bead in 2 ml of low glucose (LG) or high glucose (HG) media (24‐well) at normoxia (NX), physioxia (PX), and hypoxia (HX). (A) Oxygen contour plots for lower animal/healthy human metabolism. (B) Oxygen contour plots for a higher degenerated phenotype. (C) Minimum oxygen concentrations in the alginate bead at steady state for the different seeding densities, metabolic rates, and nutrient conditions.

**FIGURE 4:**
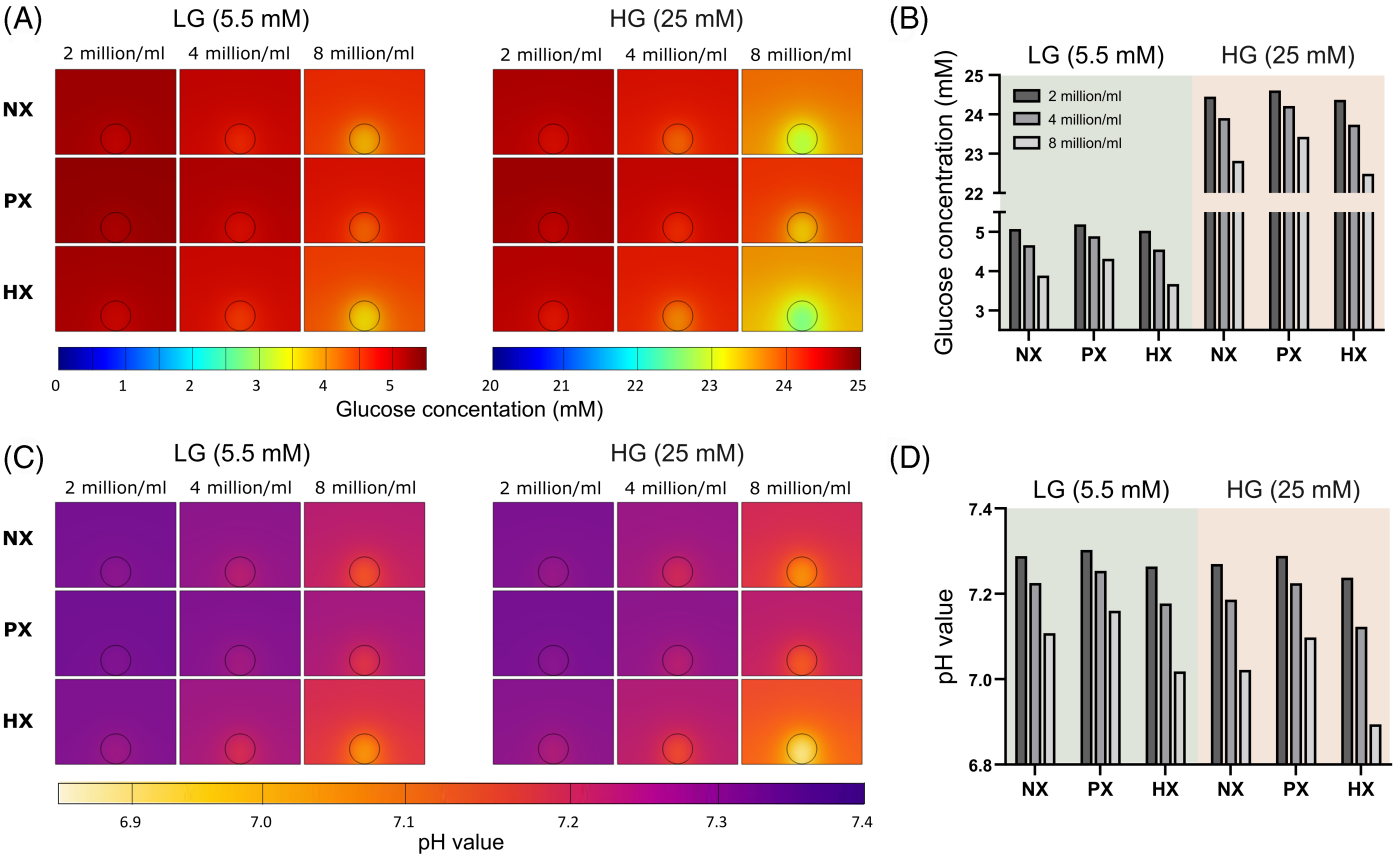
(A) Contour plots showing the glucose concentration in a single alginate bead of different seeding densities in low glucose (LG) and high glucose (HG) media, just prior to a media exchange, at normoxia (NX), physioxia (PX), and hypoxia (HX). (B) Corresponding minimum glucose concentrations within the alginate bead prior to media exchange. (C) Contour plots showing the pH level in a single alginate bead of different seeding densities in LG and HG media, just prior to a media exchange, at NX, PX, and HX. (D) Corresponding minimum pH levels within the alginate bead prior to media exchange.

Figure 3 presents the oxygen gradients through a single alginate bead (30 μl) cultured in 2 ml of media in a 24‐well plate, while Figure 4 presents the corresponding glucose and pH results for all glucose and oxygen conditions. Like the 2D models, a transient analysis was performed incorporating a regular feeding schedule of twice weekly. Subsequently the results are presented at day 3, just prior to a media exchange, to capture the minimum concentrations established within the alginate bead. Naturally the oxygen gradients will stabilize more quickly, but nonetheless they are presented at this timepoint for consistency. For cells with lower rates of oxygen metabolism (Figure 3A), oxygen does not appear to reduce significantly within a bead of 2 or 4 million cells/ml (16.4 or 14.4 %O_2_ at NX and LG), while at 8 million cells/ml oxygen drops to 11.1 %O_2_. As expected, the reduction in oxygen within beads cultured in HG is less due to the slightly lower OCR under these conditions. However, even at NX conditions, cells with very high rates of oxygen metabolism (Figure 3B), predict minimum oxygen levels of 11.3 %O_2_, 5.9 %O_2_, and 1.4 %O_2_ within beads with 2, 4, and 8 million cells/ml, respectively. In Figure 3C the minimum oxygen concentrations predicted within the beads can be compared across both OCR, for all seeding densities and external boundary concentrations. Similarly, Figure 4A,B present the glucose gradients within the culture well and minimum bead concentrations for the different seeding densities and external boundary conditions. Glucose appears in excess at HG and even at LG conditions with 8 million cells/ml, glucose does not fall critically low between media exchanges. Nonetheless, under HG conditions glycolysis is not rate limited by low glucose (<5 mM) and as a result pH is predicted to drop lower within the beads cultured at HG compared with LG (Figure 4C). This is most apparent in Figure 4D under HX conditions, where glycolytic rates are modeled to be highest. A bead with 8 million cells/ml is predicted to have a minimum pH of ~7.0 under LG conditions compared with ~6.9 under HG conditions, just prior to media exchange.

**FIGURE 5:**
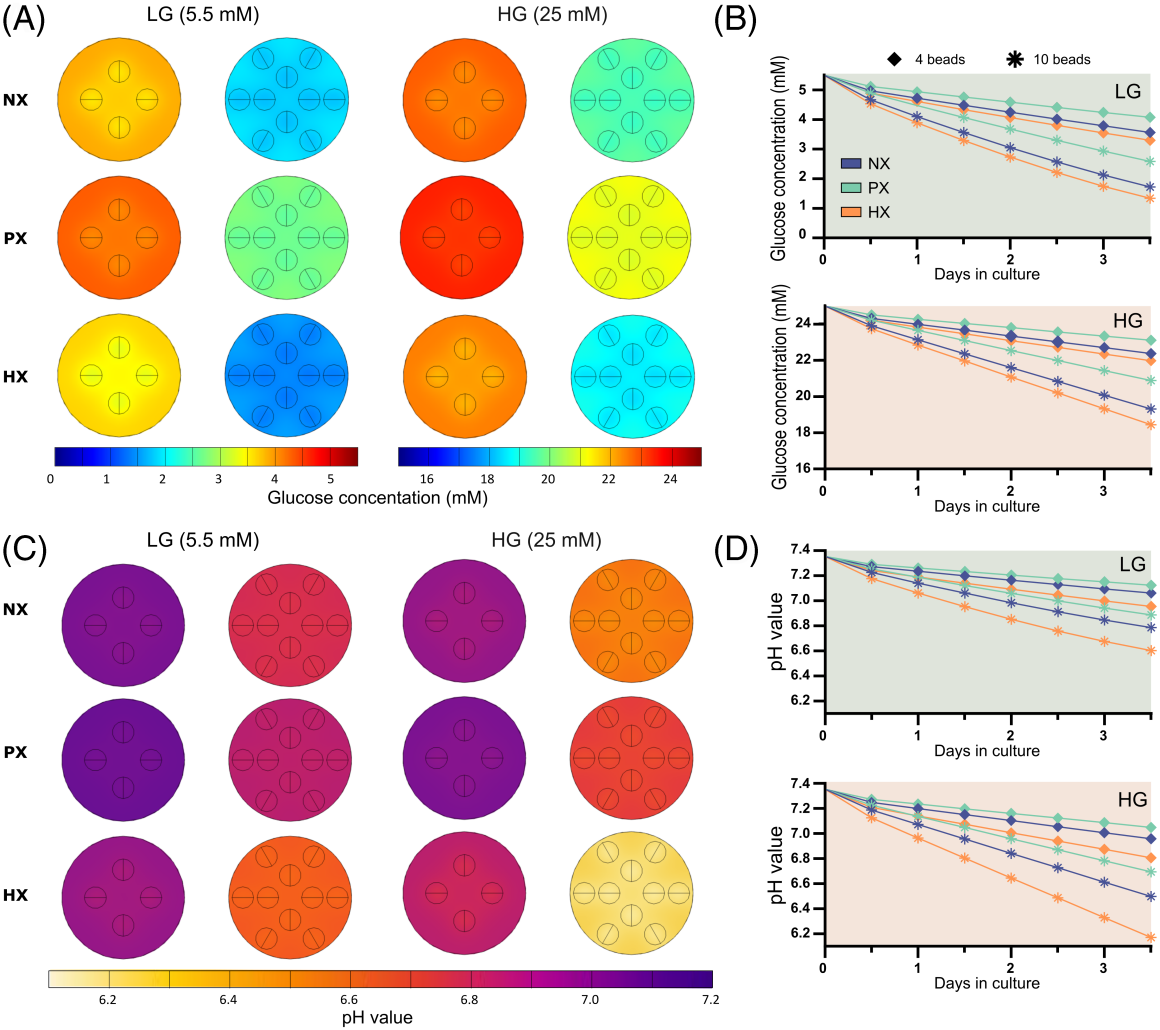
Investigating the effect of multiple alginate beads (4 or 10 beads of 4 million cells/ml) in a single culture vessel (12‐well). (A) Contour plots showing the glucose concentration in a transverse plane through the center of the beads in low glucose (LG) and high glucose (HG) media, just prior to a media exchange, at normoxia (NX), physioxia (PX), and hypoxia (HX). (B) Transient analysis of the minimum glucose concentration within an arbitrary bead within the 4 or 10 bead configurations up until the media exchange, for both LG and HG media. (C) Contour plots showing the pH in a transverse plane through the center of the beads in LG and HG media, just prior to a media exchange, at NX, PX, and HX. (D) Transient analysis of the minimum pH level within an arbitrary bead within the 4 or 10 bead configurations up until the media exchange, for both LG and HG media.

Figure 5 compares the effect of culturing 4 or 10 beads with 4 million cells/ml in a 12‐well plate. This is a transient analysis looking at the depletion of a finite supply of glucose and the build‐up of acidity between media exchanges. As a result, oxygen is not shown as it is readily available to the media surface. Figure 5A presents the glucose gradient through the center of beads just prior to a media exchange. Concentrations were observed to drop substantially lower in the well containing 10 beads at both LG and HG. In Figure 5B we see the beginning of a plateauing effect of glucose limited glycolysis in LG but not HG. Figure 5C presents the corresponding pH gradient through the alginate beads. As expected, we see significantly lower pH levels within one of the 10 beads under HX conditions and HG media. Again, in Figure 5D we see the strong effect of glycolysis not being rate limited by glucose under HG culture.

**FIGURE 6:**
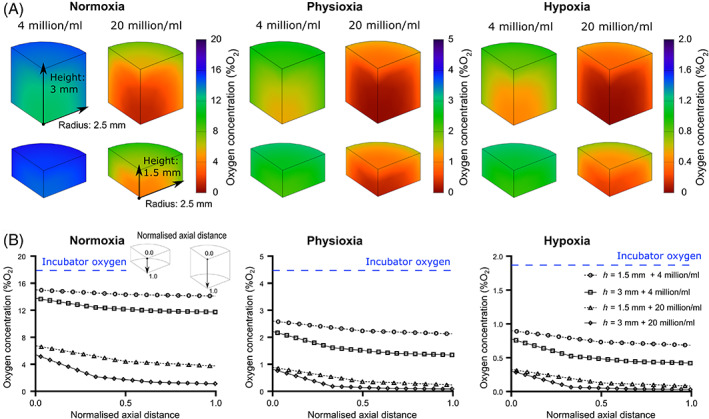
(A) Oxygen gradients through a quadrant of a cylindrical construct (radius: 2.5 mm, height: 3 mm, or 1.5 mm) containing a seeding density of either 4 or 20 million cells/ml and cultured at normoxia, physioxia or hypoxia. Cells are assumed to have the lower animal metabolic rates. (B) Corresponding axial profile of oxygen through the constructs. As indicated, the axial profile runs from the top surface of the hydrogel to the base at the bottom of the culture plate and is normalized to account for investigating two different construct heights.

**FIGURE 7:**
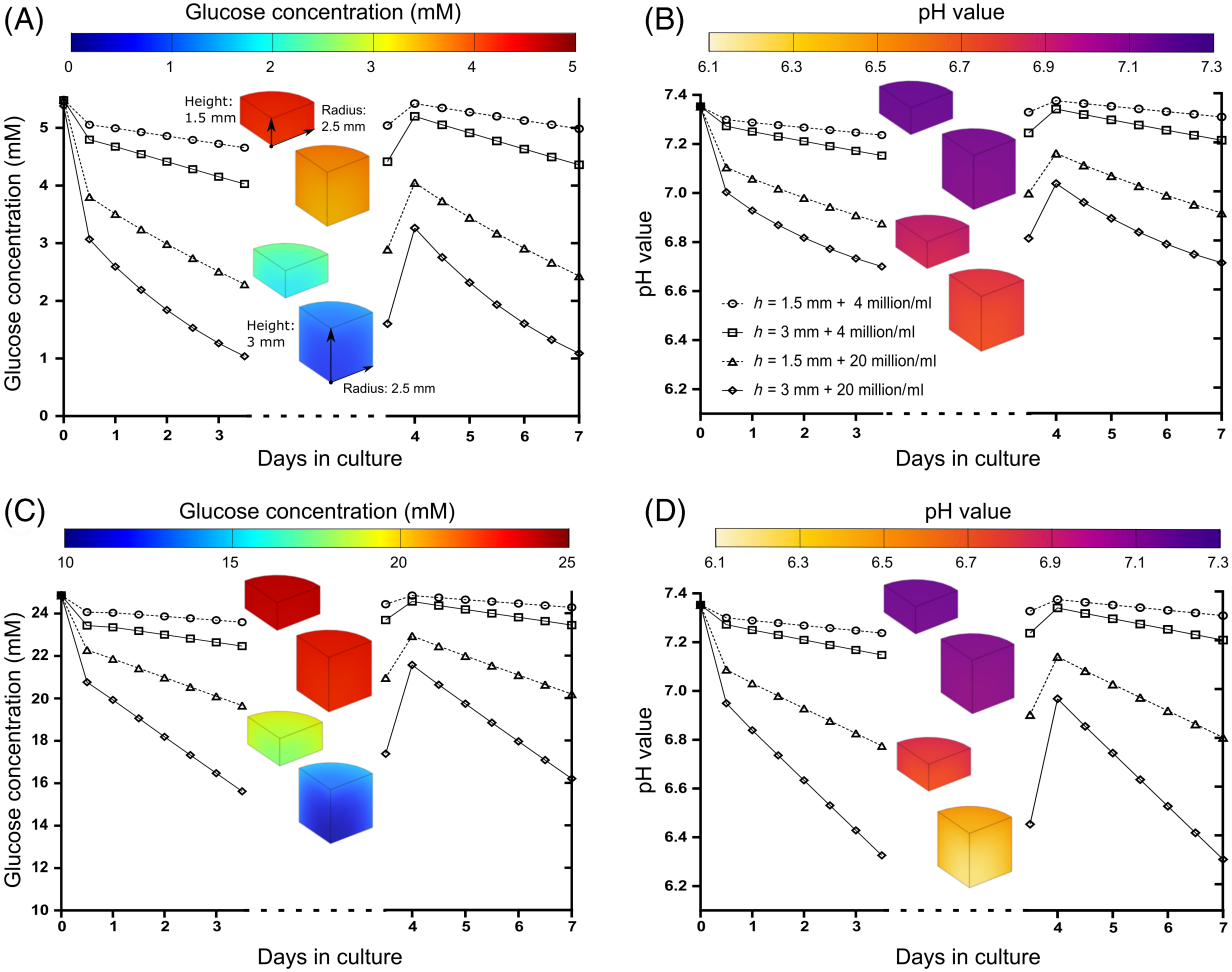
Transient analysis of glucose concentration and pH values within a cylindrical construct (radius: 2.5 mm, height: 3 mm, or 1.5 mm) containing a seeding density of either 4 or 20 million cells/ml with a media exchange performed at the midpoint. The graph represents the average values within the hydrogel and the inset contour plot represents the gradient just prior to media refresh. (A) Glucose concentrations and (B) pH values under low glucose (5.5 mM) media. (C) Glucose concentrations and (D) pH values under high glucose (25 mM) media.

Figure 6A presents the oxygen gradients through a quadrant of a cylindrical construct, comparing the effect of construct height and cell seeding density across a NX, PX, and HX incubator. Concentrations within a hydrogel containing 20 million cells/ml are significantly lower than those predicted in a 4 million cells/ml hydrogel, while a reduced construct thickness slightly alleviates the reduction in oxygen. Figure 6B compares the axial profile through the center of the cylindrical hydrogel. For example, taking the thicker construct (height = 3 mm) and 4 million cells/ml, oxygen concentrations drop to ~11.9 %O_2_ at NX, ~ 1.4 %O_2_ at PX, and 0.4 %O_2_ at HX, while the same construct with 20 million cells/ml is predicted to drop below 2 %O_2_ even at NX conditions and below 0.1 %O_2_ at PX and HX conditions. Figure 7 shows a transient analysis of the glucose and pH levels within the same constructs over the 7‐day period with a media exchange at the midpoint.

Figure 7A,B graph the average value of glucose and pH within the constructs cultured in LG, while Figure 7C,D graph the average value of glucose and pH within the constructs cultured in HG. The contour plots show the gradients of glucose and pH through the constructs just prior to the media exchange and as expected the glucose and pH increase within the hydrogel following media refresh. We see that all constructs with 4 million cells/ml remain above critical levels of glucose and pH under all culture conditions, with a twice weekly media exchange. However, thicker constructs with 20 million cells/ml start to reach critical levels of glucose and an acidic pH after just 2 days of culture under LG conditions. Furthermore, even the thinner construct with 20 million cells/ml is predicted to become acidic prior to the media refresh in HG conditions, with the thicker construct dropping to critically low levels of pH.

**FIGURE 8:**
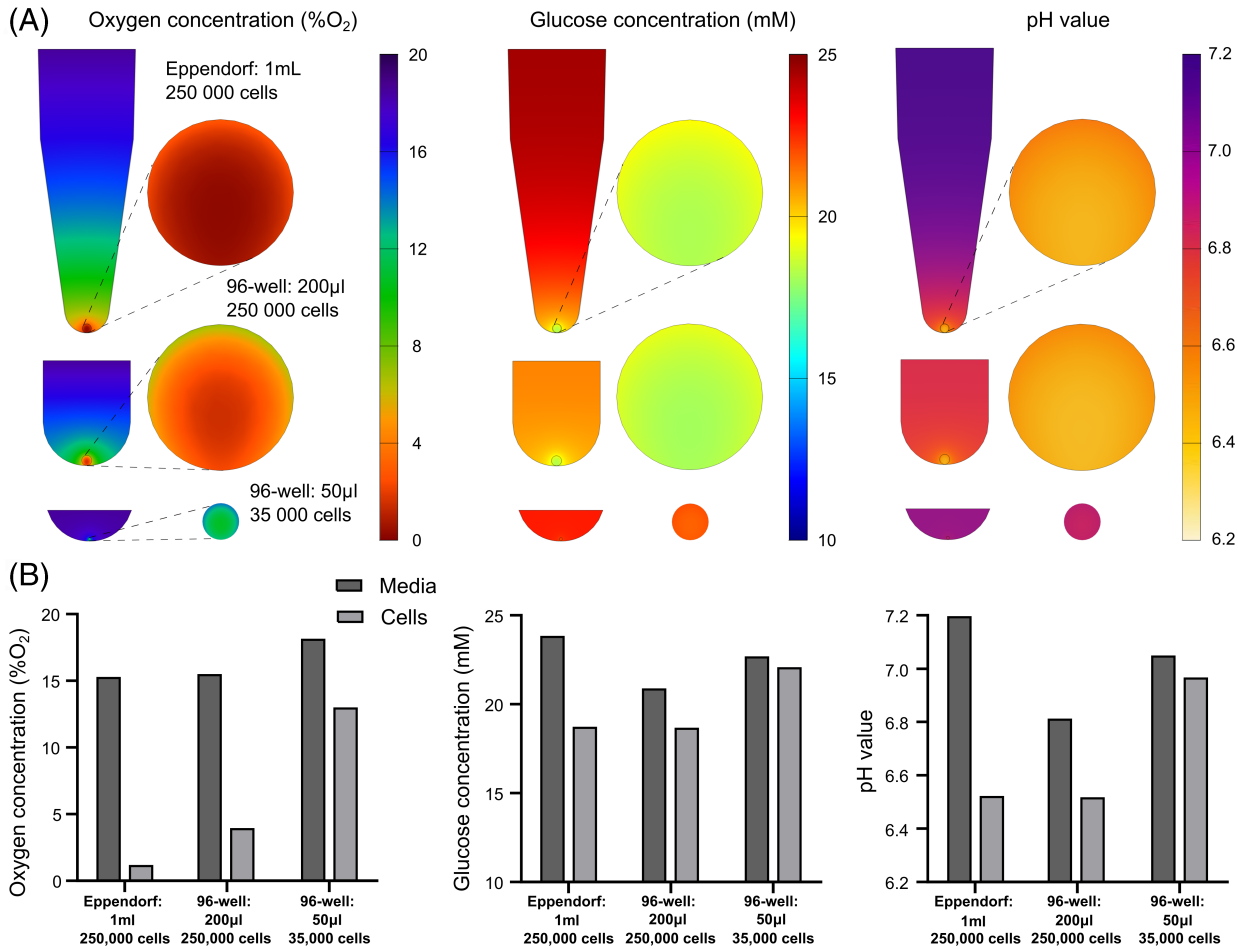
(A) Contour plots of oxygen, glucose and pH gradients through the culture media and cell aggregate of a 250 000‐cell pellet in 1 ml (Eppendorf) or 200 μl (96‐well) of media and a 35 000‐cell microaggregate in 50 μl (96‐well) of media. The presented values are predicted just prior to media refresh, under a twice weekly feeding regime. (B) Corresponding values for the average concentration of oxygen, glucose, and pH in the media and the cell aggregate prior to a media exchange in each of the three culture configurations.

Figure 8 presents the results for pellet culture. Two different types of pellet culture were investigated: conventional pellet culture of ~250 000 cells and more recently established micro‐aggregate models of ~35 000 cells. Pellet cultures were modeled either cultured in an Eppendorf with 1 ml of media or in a 96‐well with 200 μl of media, while micro‐aggregates were modeled in a 96‐well with 50 μl of media. Figure 8A presents the gradients of metabolites in the mid‐plane through the culture system on day 3, just prior to a standard media exchange. Figure 8B presents the average concentration across the culture media and within the cell aggregates at the corresponding timepoint. For oxygen, we predicted that even at NX conditions the concentration within the pellet drops down to almost 1 %O_2_ when cultured in an Eppendorf. When culturing in a 96‐well with reduced media height for oxygen diffusion, the oxygen is predicted to be ~4 %O_2_. In addition, using smaller pellets or micro‐aggregates reduces the oxygen demand and produces quite a constant gradient throughout the cellular mass with the average concentration increasing to ~13 %O_2_, only ~5 %O_2_ lower than the external boundary condition. For glucose, we see almost a 25% reduction in the concentration within the standard pellet from the initial media concentration (25 mM) and we predict no difference in pellet concentration even when the media volume is reduced from 1 ml to 200 μl, as HG still provides a surplus of glucose when performing a media exchange twice weekly. The micro‐aggregate predicts a concentration very close to the media concentration, with <3 mM reduction in media concentration prior to media exchange. However, pH drops substantially within the conventional pellets (~6.5). Although a reduced media volume will intensify the effect of lactate accumulation and the resulting pH of the media, the acidity within the conventional pellet remained similar in both configurations. The media of the micro‐aggregate is predicted to stay just above pH 7 while the cell aggregate itself drops just below pH 7 with a twice weekly media exchange.

## 4 DISCUSSION

IVD research has been challenged with heterogenous results in term of the regenerative potential and matrix synthesis of promising new therapies or treatments for disc degeneration. Consequently, harmony and standardization are currently a hot topic across the research field to advance reproducibility and accelerate clinical translation. The overall aim of this work was  to characterize the local nutrient microenvironment of 2D cell monolayers and commonly used 3D in vitro culture systems, elucidating discrepancies in nutrition between these systems and to ascertain their physiological relevance.

Looking first at 2D cell culture, the majority of NP cell culture work report 6‐well plates, followed by T‐25 flasks. This appears to be more typical in studies working with species such as rat and human,^15^, ^51^, ^52^, ^53^ possibly due to limited tissue availability and lower cell yields, while groups working with bovine and porcine cells were more likely to report larger T‐75 and T‐175 flasks.^17^ Favorably, this work predicted that the choice of culture vessels does not have a significant effect on the metabolite concentrations during monolayer culture. The greatest effect is the total cell number within the culture vessel, providing a microenvironment which changes with time due to proliferation kinetics. As the cells multiply, the rate at which glucose reduces and lactate accumulates increase. Despite this, standard media volumes together with twice weekly media exchanges are predicted to be sufficient, with glucose exhaustion and lactate acid build‐up of no significant concern, even at high levels of confluency. However, the drop in glucose and increase in pH between media exchanges will be dependent on how metabolically active the cells are and their specific glycolytic rates. This is highlighted in the case of oxygen, by comparing the “lower” animal and healthy human rates to the “higher” degenerated human rates of OCR available from the literature. While there is no major difference in the oxygen at the cell surface over time for cells with a lower OCR rate, the local cellular oxygen concentration does change over time for cells with higher rates of respiration and may need to be considered in highly oxygen dependent studies.

Oxygen is a key parameter in cell culture, as its diffusion and delivery to cells in vitro is very different to hemoglobin transportation in vivo.^26^, ^28^, ^29^, ^50^, ^54^ The level experienced by the cells reflects a balance of oxygen diffusion through the media from the surrounding incubator oxygen and OCR together with the total number of cells. While this reduction in oxygen may not be significant at NX levels, the oxygen gradient may still need to be considered if culturing particularly active cells under PX and HX conditions. Diffusion through the tissue culture plastic and flask filters was neglected, with only the media geometry modeled. This assumption was justified by diffusivity through typical culture plastic being ~4 orders of magnitude slower than media, while diffusion through the air in the filter is so rapid such that a boundary condition at the media surface is sufficient.^55^

Several studies across a number of different research areas have attempted to measure oxygen concentrations within monolayer cultures.^56^, ^57^, ^58^, ^59^, ^60^, ^61^ A study using cardiac rat cells under NX conditions measured oxygen concentrations as low as ~14.9 %O_2_ at the confluent cell surface using an oxygen glass microsensor.^61^ Another study investigating neutrophils under 4 %O_2_ external oxygen measured concentrations of <1 %O_2_ at the cell surface using an OxoProbe.^60^ Furthermore, temporal experimental measurements support the transient results in this work, with a study investigating dermal fibroblasts reporting concentrations of ~13.7 %O_2_ at 10 000 cells/cm^2^ and ~10.4 %O_2_ at a density of 90 000 cells/cm^2^.^56^ Although these cell types may be conditioned to very different microenvironmental niches and as a result have distinctly different metabolic rates compared with disc cells, they highlight that if not predicted or measured, the local cellular oxygen concentration may deviate substantially from the external oxygen boundary condition of the gas phase.^58^

When moving toward 3D cell culture systems, the majority of disc research groups performing alginate bead culture use a 30 μl bead.^62^, ^63^, ^64^, ^65^, ^66^, ^67^, ^68^ Although the current work did not predict large oxygen gradients within the beads themselves, the minimum concentrations were heavily dependent on cell number and external concentrations. Furthermore, external oxygen concentrations of NX, PX, and HX are used among these studies, thus making the local oxygen concentration a confounding factor, and not allowing for a true comparison of results between studies. Comparing different seeding densities demonstrates that cell numbers must be considered very carefully and that it is challenging to compare between samples with varying cell densities without considering that the local microenvironments will be different. When culturing a single bead in a well, we predicted that there remains a surplus of glucose between feeds, thus questioning the use of supraphysiological HG media, something which has already been experimentally examined at the larger scale of ex vivo disc organ culture.^25^ In addition, research groups have reported culturing multiple beads in a single well.^67^, ^69^, ^70^ In this scenario, we predict that more frequent media exchanges may be necessary to replenish glucose (particularly in the case of 10 beads in LG) and to circumvent the detrimental accumulation of lactate and subsequent drop in pH. The effect of refreshing the media daily is presented in Figure S2A–D and demonstrates that doing this can maintain a relatively constant level of glucose and pH throughout the culture period.

The second most popular 3D culture system are cylindrical hydrogel constructs, with the majority of studies using bovine,^71^, ^72^ porcine,^17^, ^73^, ^74^, ^75^ and goat cells.^76^ As a result, only the “lower” animal metabolic rates were modeled. In terms of the geometry, construct diameters are typically 4 or 5 mm, while the thickness appears to range from 1.5 to 3 mm.^71^, ^72^, ^74^, ^76^, ^77^ The results showed that reducing the construct thickness from 3 to 1.5 mm only slightly alleviates the nutrient demands, while cell seeding density and external boundary conditions remain the driving forces for determining the local microenvironment. Although the current work focused on comparing the thicknesses typically used in the disc field, a previous cartilage study has shown greater effects of construct size on the oxygen gradient when investigating constructs up to a diameter of 8 mm and a thickness of 4.5 mm.^78^

In order to provide confidence in the in silico construct models we can look toward a recent cartilage study that measured oxygen levels of 3.0–7.6 %O_2_ (depending on cell type) in the center of hydrogel constructs (diameter = 5 mm, thickness = 3 mm, and 20 million cell/ml) cultured at external NX conditions.^79^ Furthermore, when comparing external boundary concentration, the authors measured central concentrations of ~4.3 %O_2_ at NX and ~1.1 %O_2_ at PX. Although the above study used different cell types (stem cells and chondrocytes) with inherently different metabolic rates and a hydrogel susceptible to greater oxygen diffusion (2% agarose), the measurements are within good agreement with our predicted concentrations for 20 million cells/ml under both NX and PX conditions. Nonetheless, these experimental measurements together with the predicted results, elucidate oxygen as the critical nutrient with respect to gradients through the constructs. Thus, in vitro cell culture results must be considered in context of the local microenvironmental niche rather than the external oxygen concentration. Alternatively, the external boundary oxygen concentration could be manipulated based on the parameters of the specific system to establish the desired physiologically relevant concentrations locally. For example, culturing high cell densities at HX or even PX levels of oxygen were predicted to yield a markedly lower oxygen concentration compared with the average in vivo human microenvironment which is believed to be closer to ~6 %O_2._ ^24^, ^80^ The results of this study suggest that high cell density constructs may be best suited to culturing at external NX conditions, while lower or more native cell densities require “PX” incubator levels in the disc field to be raised from ~5 to ~10 %O_2_ to create more physiologically relevant oxygen niches, in an attempt to reduce the heterogeneity across studies in terms of the oxygen environment.

Like the alginate bead, the cylindrical hydrogels also bring into question the physiological relevance of culturing in HG, particularly when assessing the suitability or effectiveness of cells as a therapy for IVD regeneration. Not only is the glucose supraphysiological for both low and high cell densities, but the models predict greater acidity in the HG constructs (particularly the 20 million cells/ml constructs). Although we did explicitly implement a significant reduction in LPR, based on experimental measurements as pH reduces below 6.7,^8^ it does not appear to be sufficient to offset the lactate accumulation. From these predictions, we speculate that construct cultures with plentiful nutrient availability (NX and HG) may still develop necrotic cores due to substantial lactic acid build‐up and that this may help explain histological results from a study by Buckley et al.^81^ The authors saw reduced collagen type II staining within the core of a chondrocyte seeded construct (15 million cell/ml) despite superior accumulation through the annulus at NX compared with throughout the whole construct at PX conditions.

Lastly, when investigating cell pellet culture and smaller micro‐aggregate systems, it is evident that the conventional pellet configuration of ~250 000 cells is not a good representative of the IVD nutrient microenvironment. The model of pellet culture was derived from the cartilage field with high cell numbers, low oxygen conditions and supraphysiological levels of glucose. Although the disc field appears to gravitate toward NX conditions, the current work highlights that despite the high external boundary concentration, the local cellular oxygen is still predicted to reach HX conditions which are not comparable to in vivo disc oxygen measurements.^24^, ^80^ HG media appears to provide a surplus of glucose even within the cell aggregate, suggesting that glucose is not the critical nutrient for viability within these configurations. However, the modeling predicts a high build‐up of lactic acid within the pellets within 3 days of culture. Therefore, we speculate that HX and acidity could be the trigger for necrotic cores and poor viability despite adequate glucose levels.

This work highlights that smaller microaggregates provide a 3D culture microenvironment which is far more tuneable, by selecting appropriate boundary concentrations and eliminating any effect of metabolite gradients. This has been readily contemplated in recent years, with the cartilage field moving toward micro‐spheroids or organoids as building blocks rather than large cartilaginous pellets associated with the restricted delivery of nutrients and the removal of waste metabolites.^82^, ^83^ Additionally, in the context of disc research, microaggregates are particularly convenient due to the relatively low cell yield of disc tissue and the limited matrix‐synthesis capacity of culture expanded NP cells.^51^, ^84^, ^85^ Recent studies have used disc cells in the range of 1000–35 000 cells per aggregate.^86^, ^87^, ^88^ Also a previous in silico model,^86^ together with the current work, suggests that NP aggregates at both ends of this range create homogeneous nutrient microenvironments, circumventing limitations such as intrinsic heterogeneity in cell phenotype and matrix synthesis due to diffusional gradients within larger pellets.

Based on the current study we recommend utilizing physiologically relevant LG media for 3D disc culture. Although this is contrary to previous findings which measured glucose concentration in “spent” HG media revealing significant drops in glucose from ~25 to ~5 mM within 3 days,^89^ it is important to note that these constructs had half the media volume of those modeled in this study and contained bone marrow derived stem cells (20 million cells/ml) stimulated by TGF‐β3 compared with growth factor free NP cells. Furthermore, Farrell et al. (2015) reported “spent” LG media dropped to ~1 mM in the absence of TGF‐β3 which is comparable to the concentrations predicted for a thicker construct containing 20 million cells/ml. This addresses the need to further elucidate differences in the metabolic profile and rates of IVD cells under additional factors such as anabolic stimulation, the presence of inflammatory cytokines or co‐culture with stem cell populations. Further experimental insight would be necessary to predict these scenarios in silico in addition to direct measurements for full characterization. Lower and more physiologically relevant glucose concentrations can be implemented in disc research by ensuring an adequate media volume and incorporating more frequent media exchanges, particularly for higher cell density cultures, to circumvent the glucose dropping below critical levels and curbing the build‐up of acidity. An example of the effect of a daily media exchange on cylindrical constructs can be found in Figure S2E–H.

This study is an idealized representation of culture microenvironments and thus is not definitive or without limitations. For example, proliferation kinetics were not considered within the 3D models. The rational for this is that the local microenvironment itself will influence the rate of proliferation and the transient analysis was only performed up until the first media exchange (~3 days). Traditional pellet cultures under both NX and PX (5 %O_2_) have shown reduced DNA content from day 1–14, while microaggregates under both conditions have exhibited a reduction from day 14 to 28 making it challenging to quantify cell proliferation within this system.^90^, ^91^ Furthermore, a species‐specific proliferation rate of cell aggregates may need to be considered.^92^ In terms of the hydrogel constructs, we assumed constant diffusion coefficients for each solute. However, substantially increasing the seeding density has been speculated to act as a form of diffusion barrier and any reduction in diffusion was not taken into account in the 20 million cell/ml models.^31^

Although we sought to capture the range of metabolic experiments on disc cells available in the literature, the rates used in the models are not without caveats. There was an assumption that rates were not influenced by culture time or biomaterial type. Jaworski et al. (2019) investigated the changes in metabolism during prolonged culture (up to 21 days) and reported differences in GCR between day 1 and day 5 at both NX and PX, with GCR significantly reducing over 21 days at NX.^13^ Additionally, stem cells used in the cartilage field have shown significantly reduced OCR after just 24 h in a pellet culture configuration and indicating a shift toward predominant glycolysis with long‐term culture. The majority of metabolism experiments on animal cells embedded freshly isolated cells in a hydrogel to maintain their 3D phenotype, justifying their use in our 3D bead and construct models.^8^, ^9^, ^10^, ^13^ However, it is likely that there are intrinsic differences in metabolic rates between 2D and 3D cell configurations which have not been taken into account in this work due to a limited availability of appropriate literature. Despite this, we do believe that by capturing a “low” and “high” OCR rate we have accounted for any large deviation which may exist between configurations, particularly with oxygen being the more limiting nutrient within 2D systems. Furthermore, rates for human cells are unfortunately limited to just one study where healthy and degenerated cells underwent identical monolayer expansion (HG + NX) for more than 3 weeks before measurement within a cell suspension.^12^ Cisewski et al. (2018) reported that degenerated cells had a significantly higher OCR (2–5 times greater) and a unique glucose response compared with healthy human cells, suggesting a distinct pathological phenotype. An alternative study looking at the bioenergetic effect of in vitro induced senescence, found senescent cells exhibited increased mitochondrial ATP‐linked respiration and taken together, results suggest a metabolic alteration is necessary to meet the degenerative energy demand for the production and secretion of inflammatory and catabolic factors.^93^

The large range in rates of disc cells reported may reflect differences in isolation procedures, expansion, measurement configuration and the difficulty of obtaining reproducible interlaboratory measurements.^57^ This large variability between studies, in addition to limited availability of literature resulted in averaged or idealized glycolytic rates (GCR + LPR) being used within the in silico models based on the external boundary conditions, rather than modeling each specific cell type. However, it has been reported that notochordal IVD cells are more metabolically active and more sensitive to nutrient deprivation.^10^ In some studies, high oxygen has been observed to reduce the rate of glycolysis which is known as a positive Pasteur effect.^20^, ^94^ However, a number of other studies have shown a negative Pasteur or no effect for NP cells.^8^, ^14^ Despite IVD cells preferring a more prevalent glycolytic pathway for energy in its harsh microenvironmental conditions, the reasons for differences in observed phenomenon under varying nutrient concentrations remain unclear. When explicitly modeling the averaged glycolytic rates (Table 1), it is apparent that a positive Pasteur is not captured throughout the compiled literature, with PX conditions appearing to have the lowest rates and thus predict the highest concentrations of glucose and pH within the 3D models. To address these potential limitations, we performed a sensitivity analysis of the lowest and highest metabolic rates (Appendix S1).

Taken together, this work highlights the importance of considering the metabolic demands of the specific cell type being utilized (animal vs. human, notochordal vs. chondrocyte‐like, healthy vs. degenerated) in conjunction with their specific culture configuration (2D and 3D). Depending on the choice of culture vessel, geometry, external concentrations, and cell seeding density, different and distinct local nutrient microenvironments will be created. Furthermore, these resulting nutrient concentration gradients have been shown to be important in regulating cell viability, gene expression, and matrix synthesis. Understanding the local cellular nutrient concentrations of in vitro culture regimes may help advance the disc field toward designing and developing more homogeneous and physiologically relevant systems.

## 5 CONCLUSION

Predominantly, the models in this work seek to illustrate the effect that parameters such as external boundary conditions and cell seeding densities have on the local nutrient microenvironment. It highlights that large variation and gradients in metabolite concentrations are easily established without careful consideration of these key parameters and that this diversity currently exists across the disc research field. As a result, we call for greater attention to the specific local microenvironment when trying to understand heterogeneity in results between studies. While one external concentration may be suitable for one culture configuration, they may not be appropriate for another. External conditions need to be tailored to the specific cells and culture system to establish homogeneous and physiologically relevant microenvironments. We believe that with more deliberate consideration of the external boundary concentrations and in vitro culture design, harmony and standardization of a physiologically relevant microenvironment will push toward greater reproducibility and more successful translation of findings across the field.

## AUTHOR CONTRIBUTIONS

Both authors contributed substantially to the conception and design of the work. Emily E. McDonnell performed the acquisition and interpretation of literature data, computational modeling, analysis presentation and interpretation of results, drafting of the article, revising it critically, and final approval. Conor T. Buckley, as the overall project funding holder, takes responsibility for the integrity of the work from inception to finalized article, provided substantial contribution to data interpretation and presentation, drafting of the article, revising it critically, and final approval.

## CONFLICT OF INTERESTS

The authors declare no conflict of interest.

## Supporting information


**Figure S1** (A) The exponential increase in total cell number within a T‐25 flask or a 6‐well plate based on our own observed population doubling time (Growth rate A) or a slower population doubling time reported by Sakai et al. where rabbit cells in a T‐25 flask reached 80% confluency in 12–15 days (Growth rate B). (B) The oxygen concentration at the cell surface over time for cells cultured at normoxia (NX) with Growth rate A and incorporating one media exchange. (C) The oxygen concentration at the cell surface over time for cells cultured at NX with Growth rate B and incorporating three media exchanges. (D) Sensitivity analysis on the effect of the lowest and highest rates of metabolism reported in the literature on the minimum oxygen, glucose and pH values in an alginate bead or hydrogel construct containing 4 million cells/ml. The dashed colored lines represent the concentrations predicted in the corresponding culture configuration using the averaged rates for the appropriate external boundary conditions (NX and LG in this case).

**Figure S2** Comparison of the effect of a standard twice weekly media exchange to a daily media refresh on the minimum glucose and pH values in a 4 and 10 bead culture at (A) low glucose (LG) and (B) high glucose (HG). Compares the effect of a standard twice weekly media exchange to a daily media refresh on the minimum glucose and pH values in a 4 million cells/ml hydrogel construct at (C) LG and (D) HG.

**Table S1** List of reviewed manuscripts and relevant experimental details extracted from the literature.

**Table S2** Oxygen consumption rates (OCR) measured at different glucose concentration for nucleus pulposus cells from a range of species and age/degeneration stages.
**Table S3.** Glucose consumption rates (nmol/million cells/h) measured at different glucose concentrations and varying oxygen levels for nucleus pulposus cells from a range of species and age/degeneration stages. Abbreviations: HX, hypoxia; NX, normoxia; PX, physioxia.
**Table S4.** Lactate production rates (nmol/million cells/h) measured at different glucose concentrations and varying oxygen levels for nucleus pulposus cells from a range of species and age/degeneration stages. Abbreviations: HX, hypoxia; NX, normoxia; PX, physioxia.
