# Supplementary figures and images for: Two‐ and three‐dimensional in vitro nucleus pulposus cultures: An in silico analysis of local nutrient microenvironments
Source: JOR Spine. 2022 Aug 30;5(3):e1222. doi: 10.1002/jsp2.1222 (PMC9520769; doi:10.1002/jsp2.1222)

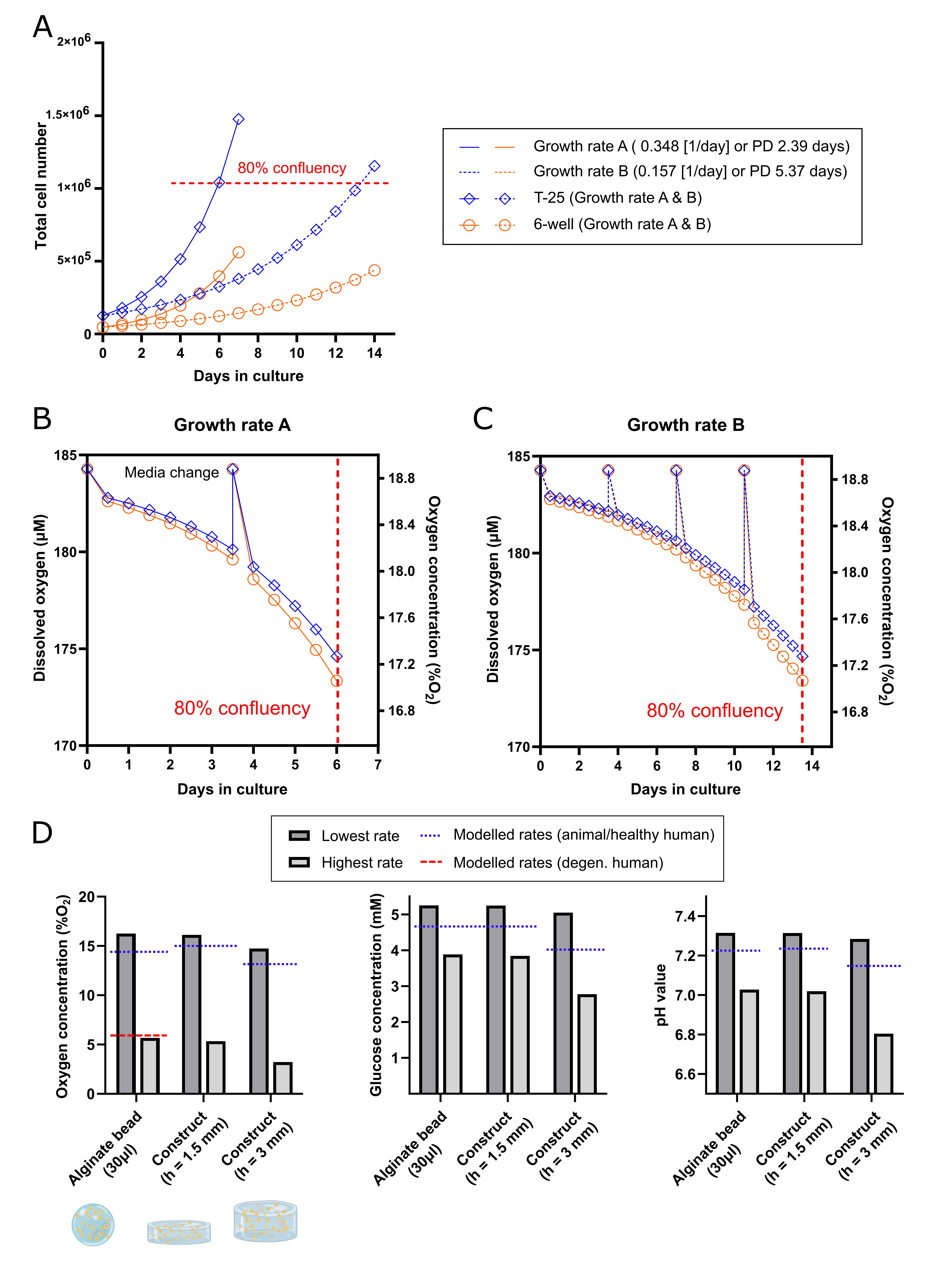

Supplement: Supplementary file 1 — Figure S1 (A) The exponential increase in total cell number within a T‐25 flask or a 6‐well plate based on our own observed population doubling time (Growth rate A) or a slower population doubling time reported by Sakai et al. where rabbit cells in a T‐25 flask reached 80% confluency in 12–15 days (Growth rate B). (B) The oxygen concentration at the cell surface over time for cells cultured at normoxia (NX) with Growth rate A and incorporating one media exchange. (C) The oxygen concentration at the cell surface over time for cells cultured at NX with Growth rate B and incorporating three media exchanges. (D) Sensitivity analysis on the effect of the lowest and highest rates of metabolism reported in the literature on the minimum oxygen, glucose and pH values in an alginate bead or hydrogel construct containing 4 million cells/ml. The dashed colored lines represent the concentrations predicted in the corresponding culture configuration using the averaged rates for the appropriate external boundary conditions (NX and LG in this case). [file JSP2-5-e1222-s003.png]

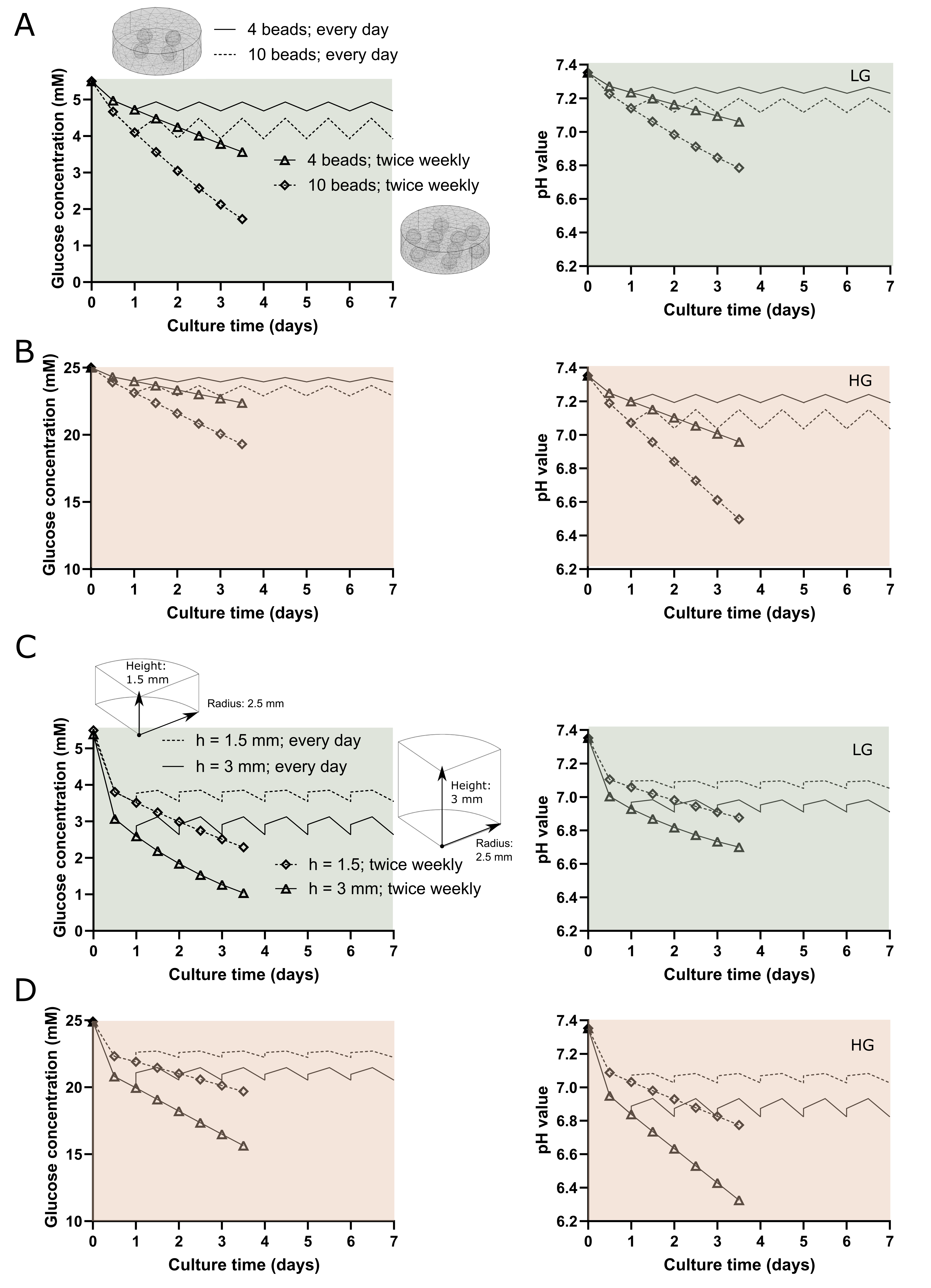

Supplement: Supplementary file 2 — Figure S2 Comparison of the effect of a standard twice weekly media exchange to a daily media refresh on the minimum glucose and pH values in a 4 and 10 bead culture at (A) low glucose (LG) and (B) high glucose (HG). Compares the effect of a standard twice weekly media exchange to a daily media refresh on the minimum glucose and pH values in a 4 million cells/ml hydrogel construct at (C) LG and (D) HG. [file JSP2-5-e1222-s002.png]
