# Supplementary material for: Two‐ and three‐dimensional in vitro nucleus pulposus cultures: An in silico analysis of local nutrient microenvironments
Source: JOR Spine. 2022 Aug 30;5(3):e1222. doi: 10.1002/jsp2.1222 (PMC9520769; doi:10.1002/jsp2.1222)
Supplement: Supplementary file 3 — Table S1 List of reviewed manuscripts and relevant experimental details extracted from the literature. [file JSP2-5-e1222-s004.pdf]

2D cell expansion

Year	DOI	First/last author	Culture flask/dish	Expansion	Media volume	Media change	Glucose	Oxygen	Species
2005	10.1097/01.brs.0000184365.28481.e3	D. Sakai/T. Hotta	-	P2	-	-	-	-	Rabbit
2006	10.1016/j.biomaterials.2005.06.038	D. Sakai/T. Hotta	T-25	12 - 15days	-	-	Low glucose (LG): 1 g/L or 5.5 mM	-	Rabbit
2008	10.1002/jor.20584	A. Hiyama/D. Sakai	-	12 - 27 days	-	Every 2 days	-	-	Canine
2008	10.1097/BRS.0b013e31817b8f53	K. Wuertz/J. Iatridis	T-25	-	-	-	LG	Normoxia (NX): 20% and Hypoxia (HX): 2%	Rat
2009	10.1186/ar2611	C. Le Maitre/J. Hoyland	T-75	Up to P2 (~ 7 days)	10 mL	-	-	-	Human
2009	10.1016/j.bbrc.2008.12.145	K. Wuertz/J. Iatridis	T-25 or 24-well plate	-	-	-	-	NX	Rat
2012	10.1007/s00586-011-1976-2	M. Peroglio/D. Eglin	96-well plate	-	100 µL	-	High glucose (HG): 4.5 g/L or 25 mM	-	Bovine
2013	10.1186/scrt331	B. Chon/J. Chen	24-well plate (with transwell insert)	P3 or P5	-	-	-	Physioxia (PX): 5%	Human
2013	10.1371/journal.pone.0075548	J. Chen/L. Setton	-	-	-	-	HG	HX	Mouse
2013	10.1155/2013/326828	C. Bucher/B. Gantenbein	-	~ 7 days	-	-	LG	-	Human
2014	10.1371/journal.pone.0099621	R. Maidhof/N. Chahine	-	P2 - P4	-	-	HG	-	Bovine
2014	10.1016/j.actbio.2013.11.013	A. Francisco/L. Setton	-	-	-	-	-	-	Porcine
2015	10.1016/j.joca.2015.02.017	K. Philips/C. Le Maitre	T-75	Up to P2	-	Twice weekly	-	-	Human
2015	10.1007/s12195-014-0373-4	P. Hwang/L. Setton	well-plate	-	-	-	-	-	Human & porcine
2015	10.1016/j.actbio.2014.10.030	D. Kim/R. Mauck	-	P2	1 mL	-	HG	-	Bovine
2015	10.1002/jor.22821	D. Gorth/L. Smith	-	P2	-	-	HG	NX	Bovine
2015	10.1016/j.joca.2014.09.012	M. Farrell/R. Mauck	-	P2	-	-	HG	NX and HX	Bovine

2015	10.22203/ecm.v029a15	D. Sakai/S. Kato	6-well plate	4 days	-	-	-	-	Human
2015	10.1089/ten.TEA.2013.0719	M. Naqvi/C. Buckley	T-75	P2	-	Every 3 days	LG	PX	Porcine
2015	10.1111/joa.12305	M. Naqvi/C. Buckley	T-175	P2	-	-	LG	NX and PX	Porcine
2015	10.1186/s13075-015-0900-2	S. Chan/B. Gantenbein	12-well plate	P2	-	-	LG	-	Human
2015	10.1097/BRS.0000000000000932	B. Walter/J. Iatridis	-	P4	-	-	HG	PX	Human
2015	10.1016/j.spinee.2015.02.003	D. Purmessur/J. Iatridis	6-well plate	P19	-	-	-	-	Human
2015	10.1186/s13075-015-0569-6	I. Arkesteijn/M. Tryfonidou	-	-	-	-	HG	PX	Canine
2016	10.1038/srep37360	H. Gilbert/J. Hoyland	6-well plate (for experiment)	Up to P4	2 mL	Every 2 - 3 days	HG	-	Human
2016	10.1055/s-0034-1376573	D. Sakai/J. Mochida	well-plate	4 days	-	-	-	-	Canine
2016	10.1097/BRS.0000000000001314	M. Naqvi/C. Buckley	T-25	-	3 mL	Every 3 days	LG	NX and PX	Porcine
2016	10.1186/s13287-016-0337-9	A. Tekari/B. Gantenbein	-	~ 7 days	-	-	-	NX and HX	Bovine
2016	10.1016/j.jbiomech.2016.02.030	M. Likhitpanichkul/J Iatridis	-	P1 - P2	-	-	HG	NX	Human
2016	10.22203/ecm.v032a08	B. Walter/J. Iatridis	-	-	-	-	HG	-	Bovine
2017	10.1016/j.actbio.2017.03.010	A. Thrope/C. Le Maitre	-	Up to P7	-	-	-	-	Human
2017	10.1016/j.actbio.2017.04.019	D. Bridgen/L. Setton	96 half-well plate	P2	-	-	-	-	Human
2017	10.1016/j.tice.2017.05.002	S. Vedicherla/C. Buckley	T-175	7 days (P1)	-	-	LG	NX and PX	Bovine
2017	10.1089/ten.TEC.2016.0355	R. May/B. Gantenbein	T-150	P2	-	Thrice weekly	LG	-	Human & bovine
2017	10.22203/eCM.v033a18	D. Rosenzweig/L. Haglund	Petri-dish (35mm; 55mm; 100mm)	~ 5 days	-	Every 3 days	HG	-	Bovine
2017	10.1038/s41598-017-17472-1	E. Krock/L. Haglund	-	P1 or P2	-	-	HG	-	Human
2017	10.1089/ten.TEA.2016.0251	F. Bach/M. Tryfonidou	-	P2	-	-	-	NX	Human & canine

2017	10.1371/journal.pone.0187831	F. Bach/M. Tryfonidou	-	P2	-	-	-	NX	Human, bovine & canine
2017	10.18632/oncotarget.21483	F. Bach/M. Tryfonidou	-	P2	-	-	-	NX	Human & canine
2018	10.1002/jsp2.1004	N. Hodson/J. Hoyland	-	Up to P4	-	Every 2 - 3 days	HG	-	Human
2018	10.1115/1.4038758	B. Shah/N. Chahine	-	P2 - P4	-	-	HG	-	Bovine
2018	10.1186/s13287-018-0797-1	R. Tang/L. Setton	6-well plate	-	-	-	-	-	Human (cell line)
2018	doi.org/10.1371/journal.pone.0202640	D. Sakai/M. Watanabe	T-25	Up to P3	-	Twice weekly	-	HX	Human
2018	10.1088/1748-605X/aaab7f	M. Naqvi/C. Buckley	-	-	-	-	-	PX	Porcine
2018	10.3390/ijms19041195	R. May/B. Gantenbein	-	P2	-	Thrice weekly	LG	HX	Human
2018	10.22203/eCM.v036a15	D. Rosenzweig/L. Haglund	-	-	-	-	HG	-	Human
2018	10.1089/ten.TEA.2017.0334	M. Cruz/J. Iatridis	-	P3 - P5	-	-	HG	NX	Bovine
2018	10.1016/j.jconrel.2018.08.019	A. Tellegen/M. Tryfonidou	24-well plate	-	-	-	HG	NX	Canine
2019	10.1002/jor.24154	B. Shah/N. Chahine	-	P2 - P4	-	-	HG	-	Human
2019	10.1096/fj.201802725RRR	B. Fearing/L. Setton	-	P3	-	-	-	-	Human
2019	10.22203/eCM.v037a09	M. Naqvi/C. Buckley	T-75	P2	-	-	LG	PX	Porcine
2019	10.3390/jcm8040433	H. Cherif/L. Haglund	6-well plate	P0 - P1	-	-	-	-	Human
2020	10.1016/j.biomaterials.2020.120057	M. Barcellona/L. Setton	-	P4	-	-	-	-	Human

2020	10.14245/ns.2040002.001	R. May/B. Gantenbein	-	-	-	-	LG	-	Human
2020	10.22203/eCM.v039a14	C. Panebianco/J. Iatridis	-	-	-	-	HG	-	Bovine

Alginate bead culture

Year	DOI	First/last author	Alginate conc.	Dimensions	Cell density	Duration	Culture vessel/media vol.	Media change	Glucose	Oxygen	Species
2005	10.1186/ar1732	C. Le Maitre/J. Hoyland	1.2%	-	1x10^6 cells/mL	-	2 mL per well	Every 2 days	-	NX	Human
2011	10.1186/ar3344	D. Purmessur/J. Iatridis	1.2%	-	2x10^6 cells/mL	4 days	12-well plate (10 beads/well); 2 mL	-	LG	PX	Porcine
2013	10.1155/2013/326828	C. Bucher/B. Gantenbein	1.2%	Vol. = 30 µl	2x10^6 cells/mL	-	-	-	HG	-	Human
2013	10.1016/j.spine.2013.05.029	M. Peroglio/S. Grad	1.2%	Vol. = 30 µl	8x10^6 cells/mL	1 week	12-well plate (4 beads per well)	-	-	PX	Human
2013	10.22203/ecm.v026a08	R. Gawri/F. Mwale	1.2%	20G needle	-	-	48-well plate (5 beads per well)	-	HG	NX	Human & bovine
2013	10.1097/BSD.0b013e31826e0ca4	R. Abbott/J. Iatridis	1.2%	-	2x10^6 cells/mL	3 weeks	12-well plate (10 beads/well); 2 mL	Twice weekly	LG	PX	Human
2014	10.1186/1471-2474-15-422	B. Gantenbein/S. Chan	1.2%	Vol. = 30 µl	4x10^6 cells/mL	1 week	-	-	HG	NX	Porcine & bovine
2015	10.1016/j.joca.2015.02.017	K. Philips/C. Le Maitre	1.2%	19G needle	2x10^6 cells/mL	2 weeks	-	Twice weekly	-	NX	Human
2015	10.1186/s13075-015-0900-2	S. Chan/B. Gantenbein	1.2%	Vol. = 30 µl	-	-	12-well plate	Thrice weekly	-	-	Human
2015	10.1097/BRS.0000000000000932	B. Walter/J. Iatridis	1.2%	-	2x10^6 cells/mL	2 weeks	12-well plate; 2 mL	-	LG	PX	Human

2015	10.1186/s13075-015-0569-6	I. Arkesteijn/M. Tryfonidou	1.2%	-	3x10^6 or 6x10^6 cells/mL	4 weeks	-	-	HG	PX	Canine
2016	10.1097/BRS.0000000000001314	M. Naqvi/C. Buckley	1.5%	Diameter (D) = 5 mm (12G needle)	4x10^6 cells/mL	-	24-well plate; 2 mL	Twice weekly	LG	NX and PX	Porcine
2016	10.22203/ecm.v032a11	F. Bach/M. Tryfonidou	1.2%	Vol. = 20 µl	3x10^6 cells/mL	4 weeks	-	-	-	PX	Bovine
2017	10.1016/j.tice.2017.05.002	S. Vedicherla/C. Buckley	-	D = 5mm	4x10^6 cells/mL	3 weeks	24-well plate; 2 mL	Twice weekly	LG	NX and PX	Bovine
2018	10.1115/1.4038758	B. Shah/N. Chahine	1.2%	21G needle	1x10^6 or 4x10^6 cells/mL	-	12-well plate (9 beads per well)	-	-	NX	Bovine
2018	10.1088/1748-605X/aaab7f	M. Naqvi/C. Buckley	1%	D = 240 µm	10x10^6 or 20x10^6 cells/mL	2 weeks	6-well plate	Twice weekly	LG	PX	Porcine
2018	10.3390/ijms19041195	R. May/B. Gantenbein	1.2%	Vol. = 30 µl	4x10^6 cells/mL	3 weeks	12-well plate	-	-	HX	Human
2019	10.1002/jor.24154	B. Shah/N. Chahine	1.2%	-	3.3x10^4 cells/bead	5 weeks	-	-	-	-	Human
2020	10.14245/ns.2040002.001	R. May/B. Gantenbein	1.2%	Vol. = 30 µl	4x10^6 cells/mL	3 weeks	-	-	-	HX	Human

Cylindrical hydrogel constructs

Year	DOI	First/last author	Hydrogel	Dimensions	Cell density	Duration	Culture vessel/media vol.	Media change	Glucose	Oxygen	Species
2008	10.1016/j.biomaterials.2007.09.018	S. Richardson/J. Hoyland	Chitosan glycerophosphate	250 µL (in transwell insert)	4x10^6 cells/mL	4 weeks	24-well plate	Every 3 days	-	NX	Human
2010	10.1002/art.27710	B. Minogue/J. Hoyland	Type I collagen	200 µL	4x10^6 cells/mL	-	24-well plate	Every 2 - 3 days	HG	-	Human
2010	10.1186/scrt18	C. Korecki/J. Iatridis	Alginate	D = 5.82 mm and h = 3.27 mm (~ 100 µl)	2x10^6 cells/mL	4 days	6-well plate (5 gels per well); 5 mL	-	HG	PX	Pocine
2013	10.1186/scrt331	B. Chon/J. Chen	Matrigel	60 µL (in transwell insert)	-	3 weeks	24-well plate	-	-	HX	Human
2015	10.1016/j.actbio.2014.10.030	D. Kim/R. Mauck	Methacrylated hyaluronic acid (HA)	D = 4 mm and height (h) = 1.5 mm	20x10^6 or 60x10^6 cells/mL	8 weeks	1 mL each	Thrice weekly	HG	-	Bovine
2015	10.1002/jor.22821	D. Gorth/L. Smith	2% agarose	D = 4 mm and h = 2.25 mm	20x10^6 cell/mL	6 weeks	1 mL each	Every 3 days	HG	NX and HX	Bovine

2015	10.1016/j.joca.2014.09.012	M. Farrell/R. Mauck	2% agarose	D = 4 mm and h = 2.25or 0.75 mm	20x10^6 cell/mL	4 weeks	-	Twice weekly	LG and HG	NX and HX	Bovine
2015	10.1089/ten.TEA.2013.0719	M. Naqvi/C. Buckley	1.5% alginate or chitosan	D = 5 mm and h = 3 mm	4x10^6 or 8x10^6 cells/mL	3 weeks	12-well plate; 2 mL	Twice weekly	-	PX	Porcine
2015	10.1111/joa.12305	M. Naqvi/C. Buckley	1.5% alginate	D = 5 mm and h = 3 mm	-	-	24-well plate; 2 mL	-	1, 5 and 25 mM	NX and PX	Porcine
2017	10.1016/j.actbio.2017.07.025	S. Gullbrand/L. Smith	7.5% oxidized dextran	D = 4 mm and h = 2.26 mm	20x10^6 cell/mL	2 weeks	-	-	HG	NX	Goat
2018	10.22203/eCM.v036a15	D. Rosenzweig/L. Haglund	2% agarose	250 µL	2x10^6 cells/mL	-	-	-	-	-	Human
2018	0.1089/ten.TEC.2017.0226	A. Krouwels/L. Creemers	Alginate, agrose, gelma, fibrin, HA-PEG & Col2	~ 50 µL	2x10^6 cells/mL	-	-	-	-	-	Human
2019	10.22203/eCM.v037a09	M. Naqvi/C. Buckley	1.5% alginate	OuterD = 9 mm, innerD = 5 mm and h = 3 mm	4x10^6 cells/mL	-	-	-	LG	PX	Porcine
2021	10.1089/ten.tea.2020.0123	H. Zlotnick/R. Mauck	Agarose	D = 4 mm and h = 2.25 mm	~ 565,000 cells (total)	8 weeks	1, 3 or 5 mL	Twice weekly	HG	-	Bovine

Cell pellets and microaggregates

Year	DOI	First/last author	Culture vessel	Cell density	Duration	Media volume	Media change	Glucose	Oxygen	Species
2010	10.1186/scrt18	C. Korecki/J. Iatridis	96-well plate	250,000 cells/pellet	-	-	Twice weekly	HG	PX	Pocine
2015	0.22203/ecm.v030a10	F. Bach/M. Tryfonidou	96-well plate	35,000 cells/microaggrecate	-	-	-	HG	NX and PX	Human, canine, porcine
2016	10.1186/s13075-016-0960-y	F. Bach/M. Tryfonidou	96-well plate	35,000 cells/microaggrecate	2 weeks	50 µL	-	HG	NX	Human & canine
2017	10.22203/eCM.v033a18	D. Rosenzweig/L. Haglund	-	1 x10^6 cells/pellet	3 weeks	-	Every 3 days	HG	-	Bovine
2017	10.1089/ten.TEA.2016.0251	F. Bach/M. Tryfonidou	96-well plate	35,000 cells/microaggrecate	4 weeks	50 µL	Twice weekly	HG	NX	Human & canine

2017	10.1371/journal.pone.0187831	F. Bach/M. Tryfonidou	96-well plate	35,000 cells/microaggrecate	4 weeks	50 µL	Twice weekly	HG	NX	Human, bovine & canine
2017	10.18632/oncotarget.21483	F. Bach/M. Tryfonidou	96-well plate	35,000 cells/microaggrecate	1 or 3 weeks	50 µL	Twice weekly	HG	NX	Human & canine
2019	10.1177/1947603519841675	S. Peck/L. Smith	-	250,000 cells/pellet	2 weeks	-	-	LG	HX	Bovine
2019	10.3390/jcm8040433	H. Cherif/L. Haglund	-	300,000 cells/pellet	-	1 mL	Every 3 days	2.25 g/L	NX	Human
