# Supplementary material for: Two‐ and three‐dimensional in vitro nucleus pulposus cultures: An in silico analysis of local nutrient microenvironments
Source: JOR Spine. 2022 Aug 30;5(3):e1222. doi: 10.1002/jsp2.1222 (PMC9520769; doi:10.1002/jsp2.1222)
Supplement: Supplementary file 4 — Table S2 Oxygen consumption rates (OCR) measured at different glucose concentration for nucleus pulposus cells from a range of species and age/degeneration stages. Table S3. Glucose consumption rates (nmol/million cells/h) measured at different glucose concentrations and varying oxygen levels for nucleus pulposus cells from a range of species and age/degeneration stages. Abbreviations: HX, hypoxia; NX, normoxia; PX, physioxia. Table S4. Lactate production rates (nmol/million cells/h) measured at different glucose concentrations and varying oxygen levels for nucleus pulposus cells from a range of species and age/degeneration stages. Abbreviations: HX, hypoxia; NX, normoxia; PX, physioxia. [file JSP2-5-e1222-s001.docx]

# Supplementary figures


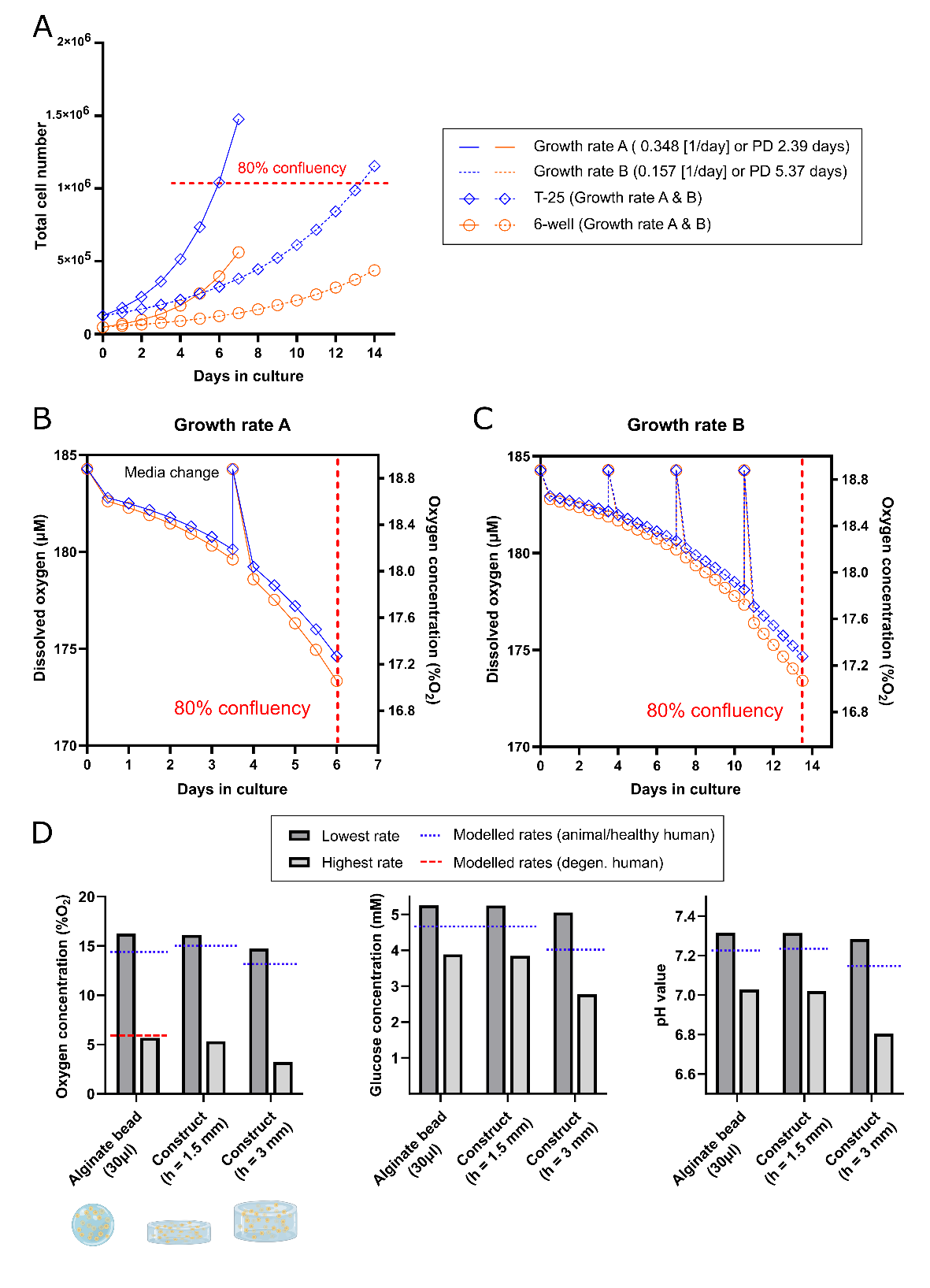


**Figure S1. (A)** The exponential increase in total cell number within a T-25 flask or a 6-well plate based on our own observed population doubling time (Growth rate A) or a slower population doubling time reported by Sakai *et al.* where rabbit cells in a T-25 flask reached 80% confluency in 12-15 days (Growth rate B). **(B)** The oxygen concentration at the cell surface over time for cells cultured at normoxia (NX) with Growth rate A and incorporating one media exchange. **(C)** The oxygen concentration at the cell surface over time for cells cultured at NX with Growth rate B and incorporating three media exchanges. **(D)** Sensitivity analysis on the effect of the lowest and highest rates of metabolism reported in the literature on the minimum oxygen, glucose and pH values in an alginate bead or hydrogel construct containing 4 million cells/mL. The dashed coloured lines represent the concentrations predicted in the corresponding culture configuration using the averaged rates for the appropriate external boundary conditions (NX and LG in this case).


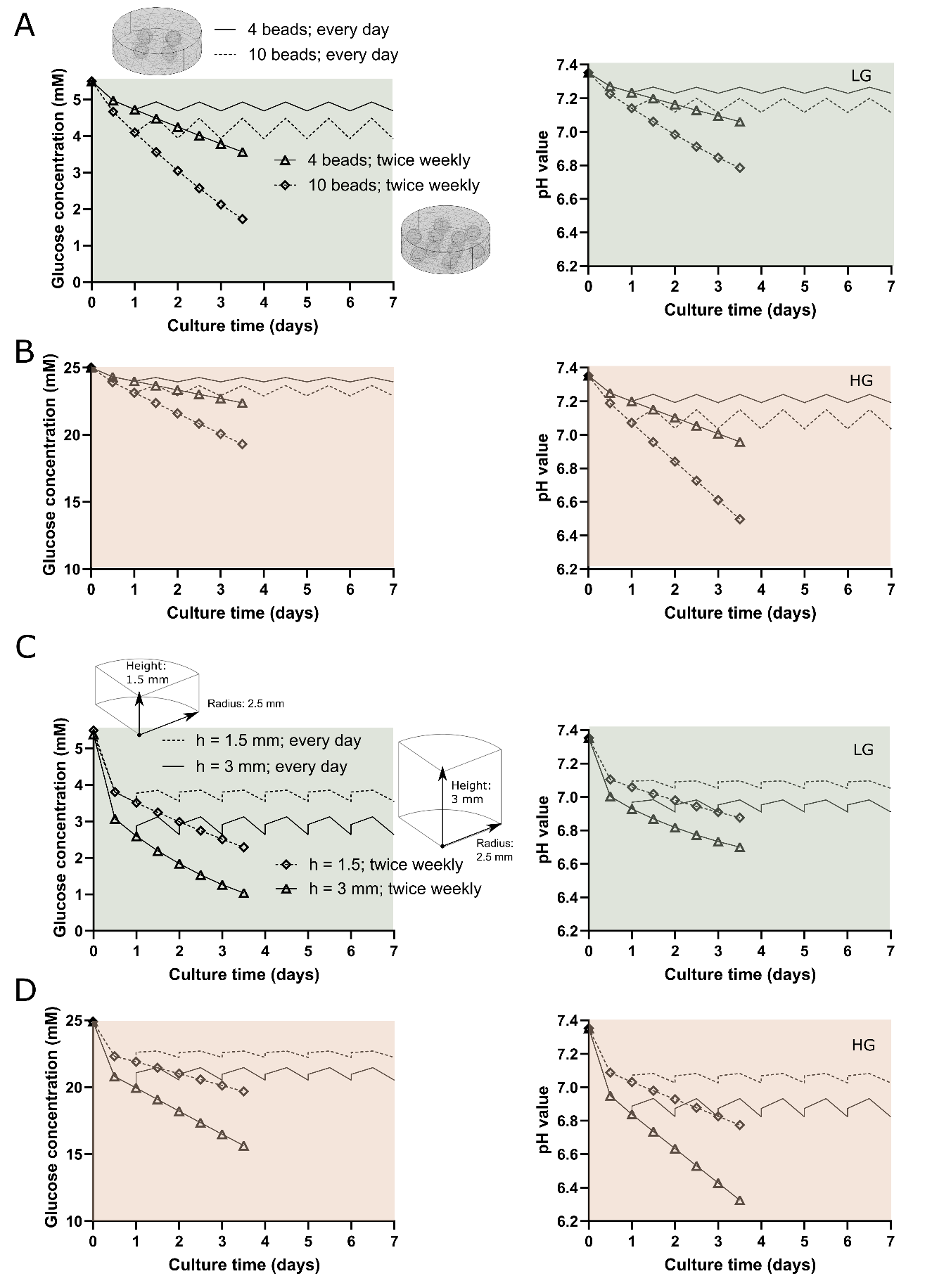


**Figure S2.** Comparison of the the effect of a standard twice weekly media exchange to a daily media refresh on the minimum glucose and pH values in a 4 and 10 bead culture at **(A)** low glucose (LG) and **(B)** high glucose (HG). Compares the effect of a standard twice weekly media exchange to a daily media refresh on the minimum glucose and pH values in a 4 million cells/mL hydrogel construct at **(C)** LG and **(D)** HG.

# Supplementary tables

**Table S1.** List of reviewed manuscripts and relevant experimental details extracted from the literature.

**(Table attached as a separate PDF file)**

Table S2. Oxygen consumption rates (OCR) measured at different glucose concentration for nucleus pulposus cells from a range of species and age/degeneration stages.

| **Glucose (mM)** | **OCR (nmol/million cells/hr)** | | | **Species** | **Age** | **Configuration** | **Reference** |
| --- | --- | --- | --- | --- | --- | --- | --- |
|  | *mean* | *std* | *n* |  |  |  |  |
| 1 | 13.43 | 7.35 | 5 | Porcine | 3–5-month-old | Agarose gel | ^9^ |
|  | 23.10 | 12.87 | 5 | Human (healthy adult) | 21 - 65 years (grade 1 or 2) | P1-P2 cell suspension | ^12^ |
|  | 95.28 | 11.02 | 5 | Human (degenerated) | 43 - 62 years (grade 3 or 4) | P1-P2 cell suspension |  |
| 2.5 | 11.59 | 3.91 | 5 | Porcine | 3–5-month-old | Agarose gel | ^9^ |
| 5 | 12.00 | 25.00 | 68 | Bovine | - | Alginate bead | ^8^ |
|  | 14.70 | 3.00 | 3 | Bovine | 18-20 months | Alginate bead | ^10^ |
|  | 60.90 | 17.00 | 3 | Porcine | 10 - 15 months | Alginate bead |  |
|  | 15.26 | 4.75 | 5 | Porcine | 3–5-month-old | Alginate bead | ^9^ |
|  | 18.06 | 12.17 | 5 | Human (healthy adult) | 21 - 65 years (grade 1 or 2) | P1-P2 cell suspension | ^12^ |
|  | 62.24 | 6.57 | 5 | Human (degenerated) | 43 - 62 years (grade 3 or 4) | P1-P2 cell suspension |  |
| 25 | 11.50 | 6.31 | 5 | Porcine | 3–5-month-old | Agarose gel | ^9^ |
|  | 13.24 | 9.41 | 5 | Human (healthy adult) | 21 - 65 years (grade 1 or 2) | P1-P2 cell suspension | ^12^ |
|  | 52.09 | 19.57 | 5 | Human (degenerated) | 43 - 62 years (grade 3 or 4) | P1-P2 cell suspension |  |

Table S3. Glucose consumption rates (nmol/million cells/hour) measured at different glucose concentrations and varying oxygen levels for nucleus pulposus cells from a range of species and age/degeneration stages. Abbreviations: NX (normoxia), PX (physioxia) and HX (hypoxia).

|  | **NX (21%)** | |  | **PX (5%)** | |  | **HX (1-2%)** | | |  |  |  |  |  |
| --- | --- | --- | --- | --- | --- | --- | --- | --- | --- | --- | --- | --- | --- | --- |
| **Glucose (mM)** | *mean* | *std* | *n* | *mean* | *std* | *n* | *mean* | *std* | *n* | | **Species** | **Age** | **Configuration** | **Reference** |
| 0.5 | 51 | 8 | 9 | 33 | 3 | 9 |  |  |  | | Porcine | 4 - 5 months | Agarose gel | ^14^ |
| 1 | 65 | 41 | 9 | 60 | 35 | 9 | 34 | 21 | 9 | | Human (healthy adult) | 21 - 65 years (grade 1 or 2) | P1 cell suspension | ^34^ |
|  | 121 | 79 | 9 | 48 | 47 | 9 | 15 | 9 | 9 | | Human (degenerated) | 43 - 62 years (grade 3 or 4) |  |  |
|  | 39 | 3 | - | 54 | 17 | - |  | - |  | | Porcine | 3 - 4 months | Alginate bead | ^17^ |
| 1.25 | 75 | 7 | 9 | 66 | 6 | 9 |  | - |  | | Porcine | 4 - 5 months | Agarose gel | ^14^ |
| 2.5 | 130 | 10 | 9 | 90 | 6 | 9 |  | - |  | |  |  |  |  |
| 3.75 | 160 | 9 | 9 | 121 | 10 | 9 |  | - |  | |  |  |  |  |
| 5 | 251 | 23 | 9 | 176 | 15 | 9 |  | - |  | |  |  |  |  |
|  | 208 | 14 | - | 250 | 13 | - | 280 | 14 | - | | Porcine | 3 - 4 months | Agarose gel | ^13^ |
|  | 124 | 102 | 9 | 160 | 109 | 9 | 68 | 29 | 9 | | Human (healthy adult) | 21 - 65 years (grade 1 or 2) | P1 cell suspension | ^34^ |
|  | 86 | 47 | 9 | 103 | 46 | 9 | 148 | 106 | 9 | | Human (degenerated) | 43 - 62 years (grade 3 or 4) |  |  |
|  | 46 | 11 | - | 111 | 11 | - |  | - |  | | Porcine | 3 - 4 months | Alginate bead | ^17^ |
|  | 107 | 13 | 6 | 253 | 74 | 6 | 74 | 10 | 6 | | Human (degenerated) | 43 - 62 years (grade 3 or 4) | P2 cell suspension | ^96^ |
|  | 95 | 14 | 3 |  | - |  |  | - |  | | Bovine | 18 - 20 months | Alginate bead | ^10^ |
|  | 205 | 28 | 3 |  | - |  |  | - |  | | Porcine | 10 - 15 months |  |  |
| 25 | 64 | 39 | 9 | 686 | 316 | 9 | 165 | 94 | 9 | | Human (healthy adult) | 21 - 65 years (grade 1 or 2) | P1 cell suspension | ^34^ |
|  | 196 | 118 | 9 | 83 | 45 | 9 | 207 | 131 | 9 | | Human (degenerated) | 43 - 62 years (grade 3 or 4) |  |  |
|  | 83 | 23 | - | 56 | 27 | - |  | - |  | | Porcine | 3 - 4 months | Alginate bead | ^17^ |
|  | 321 | 39 | - |  | - |  |  | - |  | | Porcine | 4 - 6 months | Agarose gel | ^35^ |

Table S4. Lactate production rates (nmol/million cells/hour) measured at different glucose concentrations and varying oxygen levels for nucleus pulposus cells from a range of species and age/degeneration stages. Abbreviations: NX (normoxia), PX (physioxia) and HX (hypoxia).

|  | **NX (21%)** | |  | **PX (5%)** | |  | **HX (1-2%)** | | |  |  |  |  |  |
| --- | --- | --- | --- | --- | --- | --- | --- | --- | --- | --- | --- | --- | --- | --- |
| **Glucose (mM)** | *mean* | *std* | *n* | *mean* | *std* | *n* | *mean* | *std* | *n* | | **Species** | **Age** | **Configuration** | **Reference** |
| 1 | 197 | 70 | 68 | 113 | 47 | 68 | 67 | 57 | 68 | | Bovine | - | Alginate bead | ^8^ |
|  | 92 | 62 | 9 | 127 | 53 | 9 | 30 | 16 | 9 | | Human (healthy adult) | 21 - 65 years (grade 1 or 2) | P1 cell suspension | ^34^ |
|  | 244 | 149 | 9 | 118 | 78 | 9 | 68 | 53 | 9 | | Human (degenerated) | 43 - 62 years (grade 3 or 4) |  |  |
| 5 | 89 | 59 | 9 | 168 | 80 | 9 | 98 | 15 | 9 | | Human (healthy adult) | 21 - 65 years (grade 1 or 2) | P1 cell suspension | ^34^ |
|  | 203 | 94 | 9 | 189 | 83 | 9 | 298 | 204 | 9 | | Human (degenerated) | 43 - 62 years (grade 3 or 4) |  |  |
|  | 69 | 5 | 6 | 229 | 18 | 6 | 125 | 17 | 6 | | Human (degenerated) | 43 - 62 years (grade 3 or 4) | P2 cell suspension | ^96^ |
|  | 197 | 70 | 68 | 113 | 47 | 68 | 67 | 57 | 68 | | Bovine | - | Alginate bead | ^8^ |
|  | 204 | 16 | 3 |  | - |  |  | - |  | | Bovine | 18 - 20 months | Alginate bead | ^10^ |
|  | 355 | 36 | 3 |  | - |  |  | - |  | | Porcine | 10 - 15 months |  |  |
|  | 363 | 32 | 3 |  | - |  | 358 | 39 | 3 | | Bovine | 18 - 20 months |  |  |
|  | 537 | 49 | 3 |  | - |  | 652 | 49 | 3 | | Porcine | 10 - 15 months |  |  |
| 25 | 67 | 31 | 9 | 72 | 32 | 9 | 239 | 111 | 9 | | Human (healthy adult) | 21 - 65 years (grade 1 or 2) | P1 cell suspension | ^34^ |
|  | 210 | 76 | 9 | 169 | 79 | 9 | 296 | 218 | 9 | | Human (degenerated) | 43 - 62 years (grade 3 or 4) |  |  |
|  | 395 | 11 | - |  | - |  |  | - |  | | Porcine | 4 - 6 months | Agarose gel | ^35^ |
